# Distinct tumor microenvironment profiles characterize regressive versus progressive anal precancers

**DOI:** 10.1016/j.isci.2026.117404

**Published:** 2026-09-21

**Authors:** Fernando Dias Goncalves Lima, Marieke E. Ijsselsteijn, Johanna F. Verkerk, Kirsten Rozemeijer, Carel J.M. van Noesel, Noel F.C.C. de Miranda, Jan M. Prins, Rosalie M. Luiten, Renske D.M. Steenbergen, Henry J.C. de Vries

**Affiliations:** 1 Amsterdam UMC, Location Vrije Universiteit Amsterdam, Department of Pathology, Boelelaan 1117, Amsterdam, the Netherlands; 2 Cancer Center Amsterdam, Imaging and Biomarkers, Amsterdam, the Netherlands; 3 Amsterdam UMC, Location University of Amsterdam, Department of Dermatology, Meibergdreef 9, Amsterdam, the Netherlands; 4 Amsterdam Institute for Immunology & Infectious Diseases (AI&I), Amsterdam, the Netherlands; 5 Leiden University Medical Center, Department of Pathology, Albinusdreef 2, 2333 ZA Leiden, the Netherlands; 6 Amsterdam UMC Location University of Amsterdam, Department of Internal Medicine, Division of Infectious Diseases, Meibergdreef 9, Amsterdam, the Netherlands; 7 Amsterdam UMC, VU University of Amsterdam, Department of Epidemiology and Data Science, Amsterdam Public Health, Meibergdreef 9, Amsterdam, the Netherlands; 8 Center for Sexual Health, Department of Infectious Diseases, Public Health Service Amsterdam, Amsterdam, the Netherlands; 9 Amsterdam Institute for Global Health and Development, Amsterdam, the Netherlands

**Keywords:** anal cancer, tumor immune microenvironment, high-grade squamous intraepithelial lesions, HSIL, human papillomavirus, HPV, human immunodeficiency virus, HIV, macrophage polarization, imaging mass cytometry, immunofluorescence

## Abstract

Oncogenesis of anal high-grade squamous intraepithelial lesions (HSIL) is highly variable. Therefore, all HSIL are ablated, leading to overtreatment and associated burden because not all lesions will progress to cancer. This exploratory study investigated differences in the tumor immune microenvironment between regressive and progressive HSIL in people living with HIV to enable a more tailored approach. Multiplex imaging mass cytometry showed more inflammation in HSIL that progressed to cancer and cancer samples, compared with HSIL that spontaneously regressed and controls. Densities of HLA-DR^+^ macrophage phenotypes were higher in regressive HSIL, while progressive HSIL showed a predominance of CD163^+^ and/or CD204^+^, HLA-DR^−^ macrophage phenotypes. Validation with immunofluorescence confirmed this phenotypical distribution. Moreover, HSIL lesions closest to progression, and HSIL in individuals with a low nadir CD4 count, showed more unfavorable macrophage profiles. These preliminary findings may support the development of biomarkers or immunotherapeutic targets, enabling more individualized treatment approaches for anal HSIL.

## Highlights

• Anal squamous intraepithelial lesions can be characterized by the immune infiltrate
• Densities of HLA-DR^+^ macrophages are higher in spontaneously regressing anal lesions
• CD163^+^ and/or CD204^+^, HLADR^−^ macrophages are higher in lesions progressing to cancer

## Graphical abstract

**Figure:**
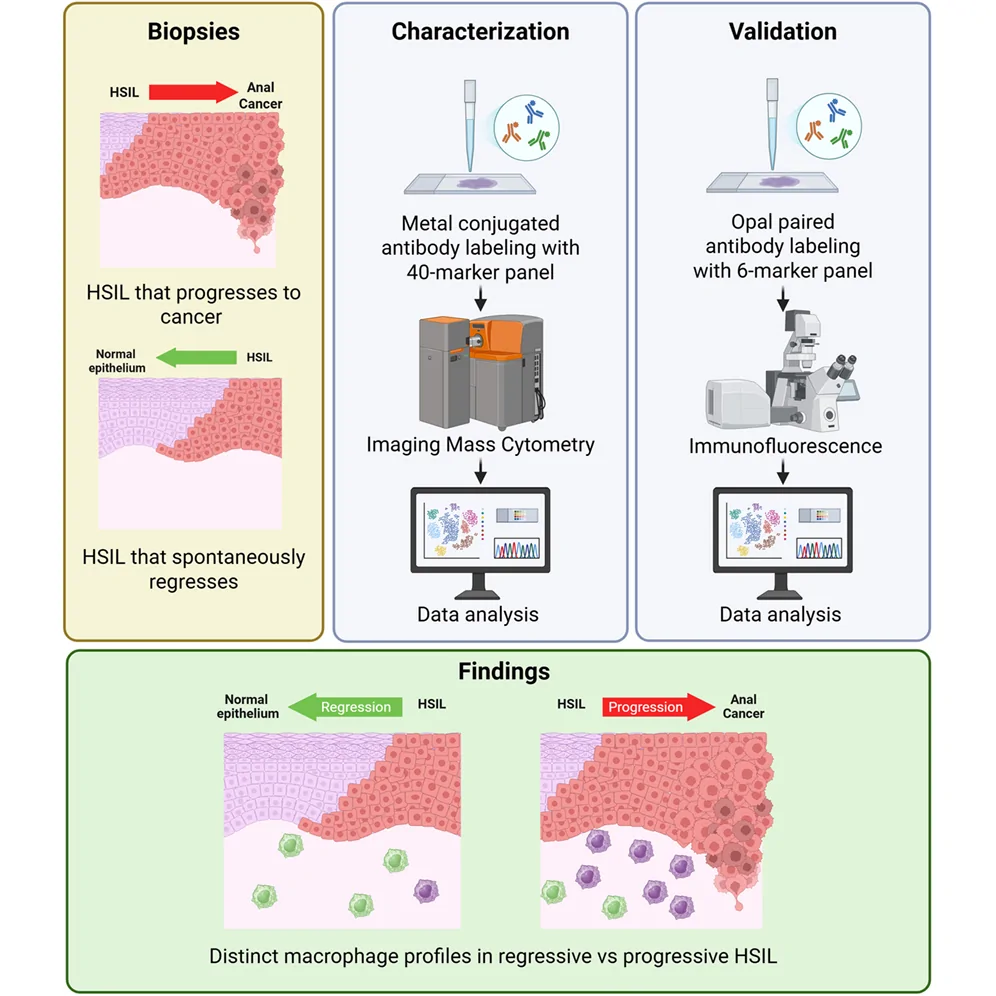

## Teaser

Cancer; Cancer systems biology; Immunology; Microenvironment

## Introduction

Human papillomavirus (HPV)-induced anal cancer is among the four most common HIV-attributable cancers, with an incidence rate of 85 per 100,000 person-years in men who have sex with men (MSM) and are living with HIV (LWH). This is 80-fold higher than in the general population^1^^,^^2^ It is associated with a high mortality rate if diagnosed in its later stages.^3^ Although early-stage anal cancer generally has a more favorable survival rate, treatment with radiotherapy and chemotherapy can lead to long-term complications and a considerable decline in quality of life.^3^^,^^4^ It is preceded by precursors called anal high-grade squamous intraepithelial lesions (HSIL), which are highly prevalent in MSMLWH (>30%).^5^ Treatment of HSIL can reduce the risk of anal cancer by approximately 57%.^6^ The lifetime progression rate of HSIL to cancer is around 10% in people living with HIV.^5^^,^^6^ Although this risk is high, most HSIL do not progress to cancer. In fact, around 30% of HSIL regress after a year without receiving any treatment.^6^^,^^7^^,^^8^ Furthermore, HSIL treatments have disappointing success rates, and the recurrence rates are high (>50%).^9^ Therefore, it is of utmost value to understand the mechanisms behind HSIL progression, including the role of the immune system.

HPV has several immune evasion strategies to persist and promote the development of precursor lesions and cancer, therefore it is important to take into account the tissue immune microenvironment.^10^^,^^11^ One major mechanism is the expression of viral oncoproteins, notably E6 and E7, which disrupt normal cell regulatory pathways and impair the function of tumor suppressor proteins, thereby indirectly affecting immune surveillance.^12^ These oncoproteins can downregulate the expression of human leukocyte antigen (HLA) molecules on the surface of infected cells, reducing the visibility of these cells to cytotoxic T lymphocytes.^13^ Additionally, HPV interferes with interferon signaling pathways, weakening the antiviral immune response and inhibiting the recruitment and activation of immune cells.^14^ The virus also creates a local immunosuppressive microenvironment by modulating cytokine profiles and promoting the accumulation of regulatory T-cells and other suppressive immune cells, like the immunosuppressive “M2-like” macrophages and myeloid-derived suppressor cells, which further diminishes the host’s ability to raise an effective immune response.^15^

For HPV-related cancers and precancers, including cervical, head and neck, vulvar, and penile cancers, components of the immune microenvironment have been proposed as biomarkers for disease progression and as targets for therapeutic interventions.^10^ For instance, a multispectral immunofluorescence biomarker panel, consisting of the sum of total of CD4^+^, CD68^+^ CD163^−^, and CD11c^+^ cells minus the total of FOXP3^+^ cells in the epithelium, had an area under the receiver operating characteristic curve (AUC) of 0.89 to predict response to imiquimod in cervical HSIL.^16^

At present, little data exist on the tumor immune microenvironment (TIME) in HPV-driven anal lesions and the potential role of TIME in the progression of anal HSIL to cancer. This study aims to characterize the TIME in both anal HSIL and cancer, with a focus on identifying the differences between HSIL lesions that progress to cancer and those that spontaneously regress. Instead of comparing the TIME across histologic AIN grades, our study uses clinical outcome (progression to cancer versus spontaneous regression), thereby capturing biological heterogeneity not fully reflected in morphologic grading. This study may therefore reveal new biomarkers for cancer risk stratification, potentially reducing the need to treat all HSIL patients, as well as identifying targets for more precise and effective therapeutic strategies.

## Results

### Imaging mass cytometry identifies 34 cellular phenotypes and a macrophage polarity shift

To identify the main immune populations in the tissue microenvironment in progressive versus regressive HSIL tissues, we employed a broad immunophenotyping panel and imaging mass cytometry to 26 biopsies (Figure 1A). This included 9 progressive HSIL, 10 regressive HSIL, 3 anal cancer, and 1 normal tissue biopsies. We identified 34 distinct cellular phenotypes, encompassing 14 myeloid, 8 lymphoid, 7 epithelial, and 7 other stromal phenotypes (Figure 1B). Additionally, we performed methylation analysis on all samples. Methylation levels were consistently high in progressive HSIL and low in regressive HSIL (*p* = 0.002, Figure 1B).

**Figure 1:**
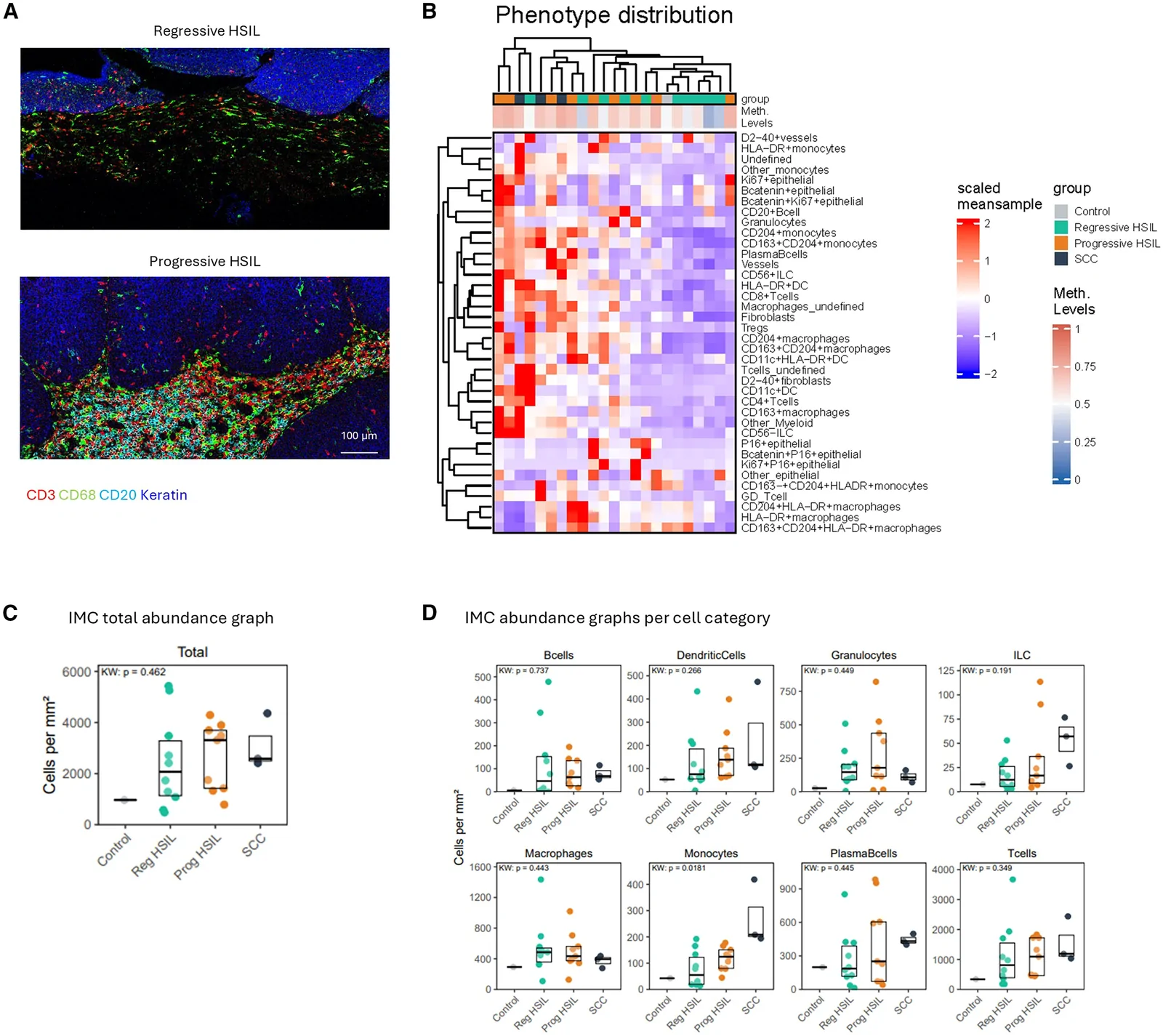
Phenotype characterization by imaging mass cytometry (A) Representative imaging mass cytometry composites from anal biopsies illustrating regressive HSIL (left) and progressive HSIL (right). Antibody channels are displayed in the colors: CD3 (T lymphocytes) in red, CD68 (macrophages) in green, CD20 (B cells) in cyan, and pan-keratin (epithelial cells) in blue. Scale bars, 100 μm. (B) Heatmap illustrating the relative abundance of immune cell phenotypes across anal biopsy samples. Each row represents a cell phenotype, and each column an individual biopsy, with columns grouped by disease category, as indicated by the color bar above the plot (Control, gray; Regressive HSIL, green; Progressive HSIL, orange; SCC, black). Absolute cell counts were normalized to tissue area (cells per mm^2^) and subsequently standardized (*Z* score) across samples; the color scale encodes relative abundance, ranging from blue (below the cohort mean) through white (mean) to red (above the mean). Rows were ordered by hierarchical clustering to emphasize similarities in phenotype distribution among samples. Additionally, methylation levels are displayed as predicted probabilities of a methylation assay using the markers *ASCL1* and *ZNF582*, ranging from blue (below cohort mean) through white (mean) to red (above the mean). Sample sizes: Control (*n* = 1), Regressive HSIL (*n* = 10), Progressive HSIL (*n* = 9), and SCC (*n* = 3). (C) Total density of immune cells across anal disease categories. Each point shows the sum of all immune cell counts normalized to tissue area (cells per mm^2^) within an individual biopsy. Point colors represent the disease categories (Control, gray; Regressive HSIL, green; Progressive HSIL, orange; SCC, black). Cross-bars mark the median, with the tops and bottoms of the bars corresponding to the first and third quartiles, respectively. Statistical comparisons: Kruskal-Wallis test and Mann-Whitney *U* pairwise comparisons. Sample sizes: Control (*n* = 1), Regressive HSIL (*n* = 10), Progressive HSIL (*n* = 9), and SCC (*n* = 3). (D) Densities of immune-cell lineages across anal disease categories. Each point shows the sum of total immune cells attributable to a given lineage within an individual biopsy. Point colors represent the disease categories (Control, gray; Regressive HSIL, green; Progressive HSIL, orange; SCC, black). Cross-bars mark the median, with the tops and bottoms of the bars corresponding to the first and third quartiles, respectively. Statistical comparisons: Kruskal-Wallis test and Mann-Whitney *U* pairwise comparisons. Sample sizes: Control (*n* = 1), Regressive HSIL (*n* = 10), Progressive HSIL (*n* = 9), and SCC (*n* = 3). DC, dendritic cells; HSIL, high-grade anal squamous intraepithelial lesions; ILC, innate lymphoid cells; KW, Kruskal-Wallis test; Progression, progressive HSIL; Regression, regressive HSIL; SCC, squamous cell carcinoma.

The total density of immune cells seemed higher in progressive HSIL and cancer samples, although not statistically significant, mostly notable in dendritic cells and monocytes (Figures 1C and 1D). Interestingly, both progressive and regressive HSIL were strongly infiltrated with macrophages (Figure 2A) and closer investigation of the specific macrophage phenotypes suggested differences in their phenotypes (Figures 2B, S1, and S2). Compared with progressive HSIL, in regressive HSIL, densities of macrophage populations that are positive for HLA-DR (CD68^+^CD163^−/+^CD204^−/+^HLA-DR^+^) seemed higher (*p* = 0.111, *p* = 0.247, and *p* = 0.430), suggesting immune stimulatory characteristics. In contrast, progressive HSIL samples suggested higher densities of stromal macrophages including immunosuppressive CD163^+^ macrophages (CD68^+^CD163^+^CD204^−^HLA-DR^-^, *p* = 0.217), CD204^+^ macrophages (CD68^+^CD163^−^CD204^+^HLA-DR^−^, *p* = 0.353), and CD163^+^CD204^+^ macrophages (CD68^+^CD163^+^CD204^+^HLA-DR^−^, *p* = 0.270).

**Figure 2:**
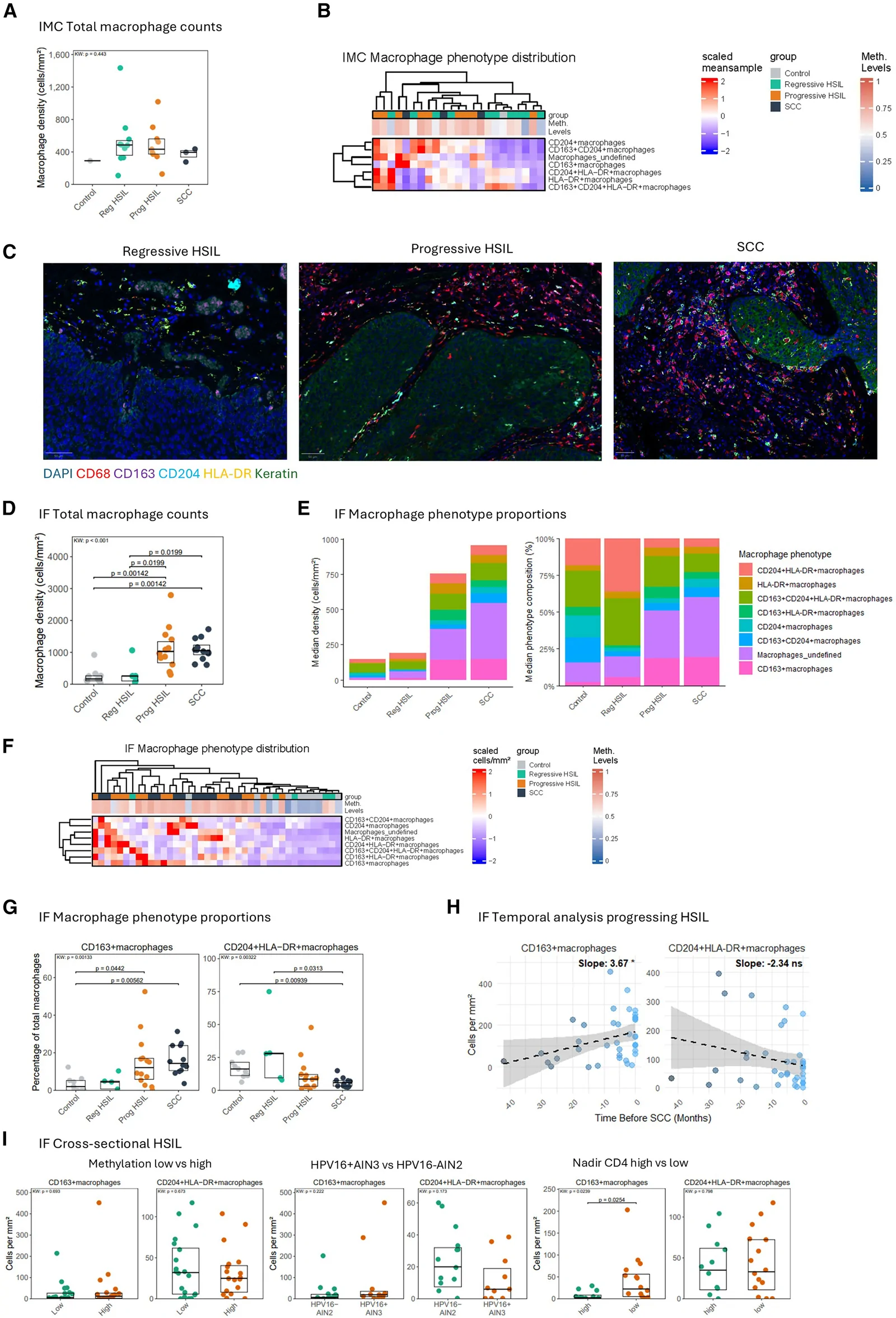
Macrophage analysis and validation (A) Total macrophage density across anal disease categories measured by imaging mass cytometry. Each point shows the sum of all macrophage phenotype counts normalized to tissue area (cells per mm^2^) within an individual biopsy. Point colors represent the disease categories (Control, gray; Regressive HSIL, green; Progressive HSIL, orange; SCC, black). Cross-bars mark the median, with the tops and bottoms of the bars corresponding to the first and third quartiles, respectively. Statistical comparisons: Kruskal-Wallis test and Mann-Whitney *U* pairwise comparisons. Sample sizes: Control (*n* = 1), Regressive HSIL (*n* = 10), Progressive HSIL (*n* = 9), SCC (*n* = 3). (B) Heatmap illustrating the relative abundance of macrophage phenotypes across anal biopsy samples, measured by imaging mass cytometry. Each row represents a macrophage phenotype, and each column an individual biopsy, with columns grouped by disease category, as indicated by the color bar above the plot (Control, gray; Regressive HSIL, green; Progressive HSIL, orange; SCC, black). Absolute cell counts were normalized to tissue area (cells per mm^2^) and subsequently standardized (*Z* score) across samples; the color scale encodes relative abundance, ranging from blue (below the cohort mean) through white (mean) to red (above the mean). Rows were ordered by hierarchical clustering to emphasize similarities in phenotype distribution among samples. Additionally, methylation levels are displayed as predicted probabilities of a methylation assay using the markers *ASCL1* and *ZNF582*, ranging from blue (below cohort mean) through white (mean) to red (above the mean). Sample sizes: Control (*n* = 1), Regressive HSIL (*n* = 10), Progressive HSIL (*n* = 9), and SCC (*n* = 3). (C) Representative immunofluorescence composites from anal biopsies illustrating regressive HSIL (left), progressive HSIL (middle), and SCC (right). Antibody channels are displayed in the colors: DAPI (cell nuclei) in blue, CD68 (macrophages) in red, CD163 (M2-like macrophages) in magenta, CD204 (activated M2-like macrophages) in cyan, HLA-DR (activated M1-like macrophages) in yellow, and pan-keratin (epithelial cells) in green. Scale bars, 50 μm. (D) Total macrophage density across anal disease categories measured by immunofluorescence. Each point shows the sum of all macrophage phenotype counts normalized to tissue area (cells per mm^2^) within an individual biopsy. Point colors represent the disease categories (Control, gray; Regressive HSIL, green; Progressive HSIL, orange; SCC, black). Cross-bars mark the median, with the tops and bottoms of the bars corresponding to the first and third quartiles, respectively. Statistical comparisons: Kruskal-Wallis test and Mann-Whitney *U* pairwise comparisons. Sample sizes: Control (*n* = 9), Reg HSIL (*n* = 5), Prog HSIL (*n* = 14), and SCC (*n* = 13). (E) Proportional composition of macrophage phenotypes. On the left, stacked bars show the median macrophage phenotype counts normalized to tissue area (cells per mm^2^) across all biopsies in each grade (ND, no dysplasia; reg, regressive; prog, progressive; SCC, squamous-cell carcinoma). On the right, stacked bars show the median proportion of each macrophage phenotype within biopsies, renormalized so that the segments for every grade sum to 100%. Sample sizes: Control (*n* = 9), Reg HSIL (*n* = 5), Prog HSIL (*n* = 14), SCC (*n* = 13). (F) Heatmap illustrating the relative abundance of macrophage phenotypes across anal biopsy samples, measured by immunofluorescence. Each row represents a macrophage phenotype, and each column an individual biopsy, with columns grouped by disease category, as indicated by the color bar above the plot (Control, gray; Regressive HSIL, green; Progressive HSIL, orange; SCC, black). Absolute cell counts were normalized to tissue area (cells per mm^2^) and subsequently standardized (*Z* score) across samples; the color scale encodes relative abundance, ranging from blue (below the cohort mean) through white (mean) to red (above the mean). Rows were ordered by hierarchical clustering to emphasize similarities in phenotype distribution among samples. Additionally, methylation levels are displayed as predicted probabilities of a methylation assay using the markers *ASCL1* and *ZNF582*, ranging from blue (below cohort mean) through white (mean) to red (above the mean). Sample sizes: Control (*n* = 9), Reg HSIL (*n* = 5), Prog HSIL (*n* = 14), and SCC (*n* = 13). (G) Proportion of the macrophage phenotypes expressing CD68^+^CD163^+^CD204^−^HLA-DR^−^ (CD163^+^macrophages) and CD68^+^CD163^−^CD204^+^HLA-DR^+^(CD204^+^HLA-DR^+^ macrophages) across anal disease categories measured by immunofluorescence. Each point shows the percentage of total macrophages attributable to a given phenotype within an individual biopsy. Point colors represent the disease categories (Control, gray; Regressive HSIL, green; Progressive HSIL, orange; SCC, black). Cross-bars mark the median, with the tops and bottoms of the bars corresponding to the first and third quartiles, respectively. Statistical comparisons: Kruskal-Wallis test and Mann-Whitney *U* pairwise comparisons. Sample sizes: Control (*n* = 9), Reg HSIL (*n* = 5), Prog HSIL (*n* = 14), and SCC (*n* = 13). (H) Temporal dynamics of macrophage subpopulations during progression from HSIL to SCC. Scatterplots show area-normalized cell densities (cells per mm^2^) for CD68^+^CD163^+^CD204^−^HLA-DR^−^(CD163^+^macrophages) and CD68^+^CD163^−^CD204^+^HLA-DR^+^(CD204^+^HLA-DR^+^ macrophages) macrophages as a function of time before SCC diagnosis; points at time = 0 correspond to SCC biopsies. Dashed lines represent the fitted linear regression, and gray shaded bands the 95% confidence intervals. Statistical comparisons: linear regression, Sample size: 26 biopsies from 14 patients. (I) Macrophage densities in cross-sectional anal HSIL samples measured by immunofluorescence. Each point shows area-normalized cell densities (cells per mm^2^) for CD68^+^CD163^+^CD204^−^HLA-DR^−^(CD163^+^macrophages) and CD68^+^CD163^−^CD204^+^HLA-DR^+^(CD204+HLA-DR^+^ macrophages) macrophages within an individual biopsy. Samples are stratified by three patient-level factors: DNA methylation status (low vs. high), histopathological grade combined with HPV genotype (HPV16^+^AIN3 vs. HPV16^−^AIN2), and nadir CD4^+^T-cell count (<200 vs. ≥200 cells/μL). Point colors represent the disease categories (Control, gray; Regressive HSIL, green; Progressive HSIL, orange; SCC, black). Cross-bars mark the median, with the tops and bottoms of the bars corresponding to the first and third quartiles, respectively. Statistical comparisons: Kruskal-Wallis test and Mann-Whitney *U* pairwise comparisons. Sample size: 62. HSIL, high-grade anal squamous intraepithelial lesions; IF, multispectral immunofluorescence; IMC, imaging mass cytometry; Prog HSIL, progressive HSIL; Reg HSIL, regressive HSIL; SCC, squamous cell carcinoma.

In contrast to the observed differences in macrophages, the T-cell compartment was more comparable between regressing and progressive HSIL samples. Interestingly, although overall densities of CD4^+^ T-cells, regulatory T-cells (T-regs), and γδ T-cells did not differ markedly across groups, markers of T-cell activation and proliferation—including PD1, Ki67, and Granzyme B seemed more frequently expressed in T-cells within regressive HSIL (non-significant, Figure S3).

Besides differences in the composition of the immune microenvironment, exploratory neighborhood analyses also suggested differences in cellular interactions. Progressive HSIL samples demonstrated enhanced interactions among macrophages, between macrophages and monocytes, and with T-cells, in particular regulatory T-cells. Conversely, regressive HSIL samples were characterized by increased contacts between T-cells and dendritic cells, as well as interactions involving HLA-DR+ macrophage phenotypes (Figure 3).

**Figure 3:**
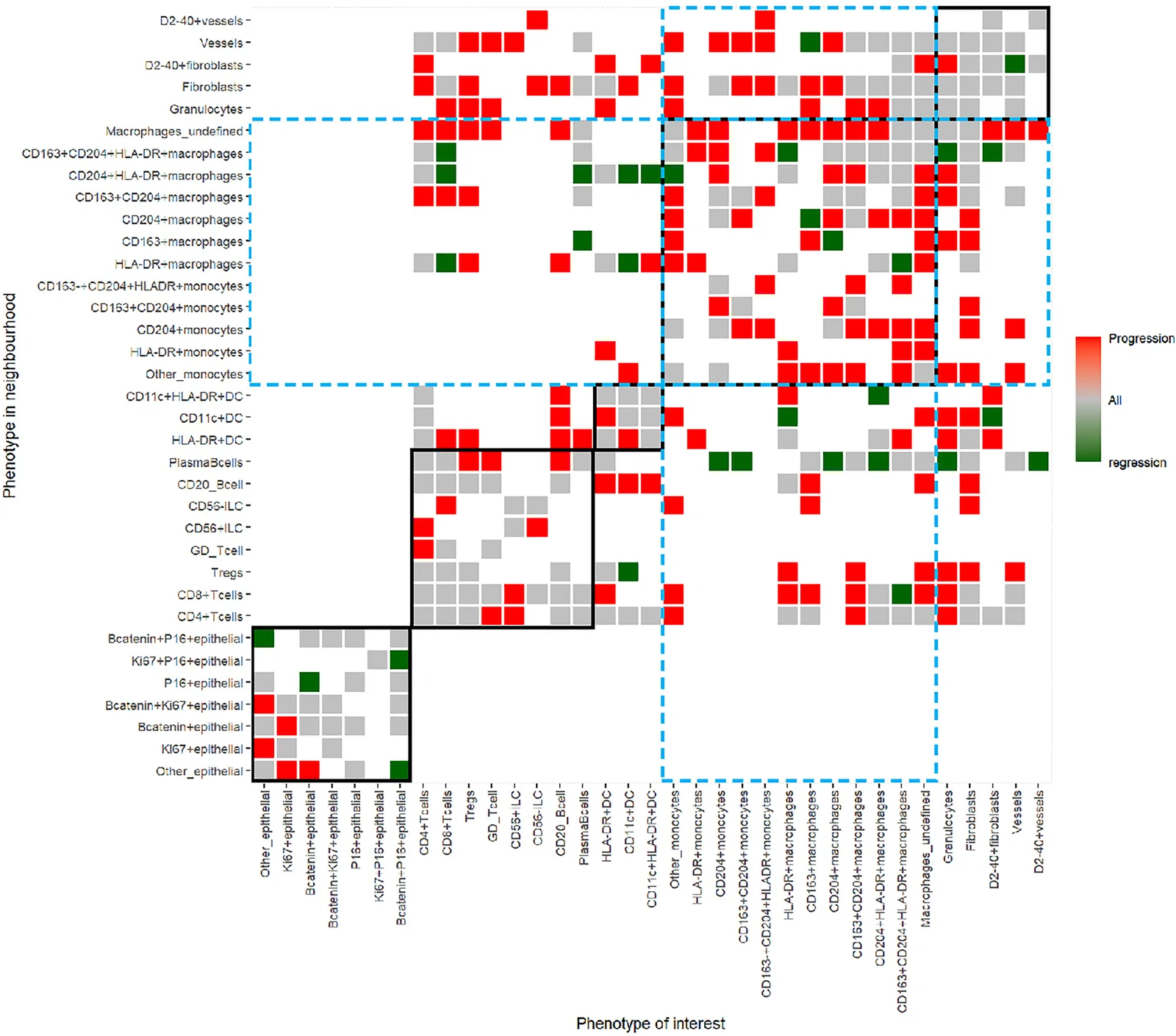
Cellular-neighborhood enrichment map in progressing and regressing HSIL Heatmap summarizing significant neighborhood enrichments between pairs of cellular phenotypes identified by imaging mass cytometry. Each tile represents an enrichment relationship between a phenotype of interest (*x* axis) and a phenotype present within its neighborhood (*y* axis). Colors indicate whether the enrichment was observed in progressing HSIL (red), regressing HSIL (green), or across all samples without outcome specificity (gray). Phenotypes are grouped into major compartments (epithelial, lymphoid, myeloid, stromal), delineated by dashed blue boxes. Solid black boxes highlight neighborhood structures that consistently differed between progressing and regressing lesions. Sample sizes: Control (*n* = 1), Regressive HSIL (*n* = 10), Progressive HSIL (*n* = 9), and SCC (*n* = 3).

### Immunofluorescence validates macrophage imbalance and suggests temporal dynamics

Given the limited statistical power of the IMC discovery cohort (*n* = 26), we validated the findings on macrophage distribution in a larger cohort using multispectral immunofluorescence microscopy (*n* = 54), investigating the distribution of CD68, CD163, CD204, and HLA-DR and Keratin to visualize the epithelial cells (Figure 2C). We analyzed 54 samples and observed that the progressive HSIL and SCC showed generally higher macrophage counts compared with the regressive HSIL and control samples (*p* < 0.020) (Figures 2D–2F and S4). Interestingly, in progressive HSIL and SCC, a significantly higher proportion of CD163^+^ macrophages was observed (*p* < 0.020). Additionally, SCC demonstrated a significantly lower proportion of CD204^+^HLA-DR^+^ macrophages compared with regressive HSIL and control samples (*p* < 0.020) (Figures 2G and S4). Sensitivity analysis excluding subjects without HIV suggested broadly similar results, though two marginal CD163^+^ macrophage contrasts lost statistical significance (Figure S5). The M2-like/M1-like macrophage proportions in the validation cohort were similar to the discovery cohort. The ratio of M2-like (CD163^+^CD204^+/−^HLA-DR^−^ and CD68^+^CD163^+/−^CD204^+^HLA-DR^−^) versus M1-like (CD68^+^CD163^+/−^CD204^+/−^HLA-DR^+^) macrophages was higher in SCC compared with regressive HSIL samples (*p* = 0.046) (Figure S6).

In stromal compartments of SCC and progressive HSIL samples, CD163^+^ macrophages and undefined macrophages (CD68^+^CD163^−^CD204^−^HLA-DR^−^) were found, often in a string-like pattern around the epithelium (Figures 2C, S7, and S8). Macrophages were generally rare in epithelial compartments, except for SCC, which had increased CD163^+^ (*p* < 0.001), CD163^+^HLA-DR^+^ (*p* = 0.047), CD204^+^ (*p* = 0.003), CD163^+^CD204^+^ (*p* = 0.017), and undefined macrophages (*p* = 0.001). Additionally, progressive HSIL had increased CD163^+^ (*p* = 0.03) and undefined macrophages (*p* = 0.011) (Figure S8).

For subjects with HSIL collected at one or more time points before progression to cancer, we performed longitudinal analyses and assessed temporal dynamics of the targeted immune cells in the process of carcinogenesis. This revealed that area-normalized counts of stromal CD68^+^CD163^+^ macrophages (*p* = 0.041), as well as epithelial populations of CD163^+^ (*p* = 0.016), CD204^+^ (*p* = 0.048), and undefined macrophages (*p* = 0.016), increased as progression to cancer approached (Figures 2H, S9, and S10). In parallel, the proportion of both stromal and epithelial macrophages that were CD68^+^CD204^+^HLA-DR^+^ decreased in proximity to progression to cancer (*p* = 0.008) (Figures S11 and S12). Altogether, there was a shift toward M2-like phenotypes as lesions neared progression to cancer (*p* = 0.012) (Figures S10A and S11). Methylation analysis of all progressive samples showed consistently high methylation levels that did not show an increase toward cancer (Figures S13–S15).

### High-risk cross-sectional HSIL recapitulate progressive signature

To further validate the findings, in the cross-sectional cohort of patients with HSIL lesions, we related marker expression with known surrogate indicators of anal cancer risk. No differences were seen between low and high methylation HSIL. HPV16^+^HSIL-AIN3 exhibited higher stromal and epithelial undefined macrophage densities compared with HPV16^−^HSIL-AIN2. Participants with a nadir CD4 cell count below 200 cells/μL had higher stromal CD163^+^ macrophage counts (*p* = 0.021) and epithelial undefined macrophage counts (*p* = 0.038), reminiscing progressive HSIL (Figures 2I, S16, and S17). AIN2 and AIN3 had higher undefined macrophage counts than controls (*p* = 0.014 and *p* = 0.0159). AIN grade did not correlate significantly with macrophage polarization of other phenotypes. This finding supports the concept that clinical outcome captures biological heterogeneity not fully reflected in morphologic grading (Figure S18).

## Discussion

To our knowledge, this is the first study to characterize the tumor immune microenvironment in anal HSIL stratified by clinical outcome (progression to invasive cancer versus spontaneous regression), possibly capturing microenvironmental variation that histological grade-based analyses may overlook. Here, we show a pattern of more CD163^+^ macrophages and relatively less CD204^+^HLADR^+^ macrophages in progressive anal HSIL and SCC than in regressing HSIL or controls in a cohort of people living with HIV. CD204^+^HLADR^+^ macrophages represent activated (M1-like) phenotypes, and CD163^+^ macrophages an immunosuppressive (M2-like) phenotype. The pattern in progressive HSIL and SCC indicates a disbalance of macrophage immune function in the local tissue immune microenvironment. The longitudinal analysis further supports this association, showing a temporal increase in CD163^+^ macrophages in HSIL that eventually progressed to cancer. Additionally, progressive HSIL and cancer samples had generally higher immune infiltration than regressive and control samples.

The two-phased approach, consisting of a characterization with imaging mass cytometry and a validation with immunofluorescence, allowed for a comprehensive phenotypic characterization of the immune cells and validation of the most distinctive markers. Using imaging techniques allowed for spatial analysis of immune cells in histological samples. Furthermore, temporal dynamics in carcinogenesis and associations with risk factors were explored. As this was an exploratory study with correlative data, no casual relationships were proven.

Similar macrophage-rich patterns associated with progression of anal HSIL have been reported in vulvar and cervical HSIL, where the influx of CD163^+^ macrophages predicts poor treatment response to imiquimod, and in HPV-driven cancers of the cervix and the head-and-neck, where abundant CD68^+^ and CD163^+^ infiltrates correlate with advanced stage and unfavorable prognosis.^16^^,^^17^^,^^18^ Mechanistically, such macrophages promote immune escape by expressing checkpoint ligands (e.g., PD-L1), recruiting regulatory T-cells, and restricting cytotoxic CD8^+^ T-cell access to tumor nests.^18^^,^^19^^,^^20^ Collectively, the degree of CD163^+^ macrophage infiltration shows promise as a progression biomarker, but confirmation in larger, prospective studies is still required.

Interestingly, we saw the same pattern of increased CD163^+^ and undefined macrophages in our cross-sectional HSIL cohort of patients with a low nadir CD4 cell count, an established risk factor for anal cancer. Saluzzo et al. propose that patients with a low nadir CD4 cell count have irreversible local depletion of CXCR3^+^CD4^+^ resident memory T-cells (Trms), despite the T-cell recovery in the blood after starting antiretroviral therapy. The depletion of Trms could lead to a Th2-like, cancer-prone environment in the anal mucosa. This shift may in turn promote recruitment and polarization of macrophages toward immunosuppressive states, contributing to immune escape and carcinogenesis.^21^ Furthermore, reduced expression of immune stimulatory cytokines in macrophages may contribute further to dysregulation of Trms.^21^^,^^22^

Expression of HLA-DR on macrophages indicates antigen-presenting, M1-like polarization, which is thought to support effective anti-HPV immune responses.^23^ IMC analysis revealed a trend toward increased macrophage phenotypes positive for HLA-DR in regressive HSIL, and immunofluorescence confirmed this for CD204^+^HLA-DR^+^ macrophages. Although immunofluorescence did not confirm this increase for HLA-DR^+^ macrophages (e.g., CD68^+^CD163^−^CD204^−^HLA-DR^+^), as this phenotype was increased in progressive HSIL, this shift did not change the overall intralesional macrophage composition. Altogether, the relative abundance of HLA-DR^+^ phenotypes proportioned against M2-like macrophages shows promise as a biomarker of regression, but larger studies are needed to confirm its utility.

Studies in cervical cancer have linked the presence of CD204^+^ macrophages to unfavorable prognostic factors like disease-free survival and metastasis.^24^^,^^25^ Although our IMC data suggest that CD204^+^ and CD163^+^CD204^+^ macrophage densities were higher in progressive HSIL, it was not confirmed with the immunofluorescence analysis. Instead, the trend toward (proportionally) decreased macrophage phenotypes positive for CD204^+^HLA-DR^+^ in progressive HSIL that was observed in both IMC and immunofluorescence. Further research is needed to clarify how CD204 expression influences anal carcinogenesis.

The total macrophage densities of progressive HSIL biopsies were higher in the validation cohort as compared with the discovery cohort, likely because the validation samples were obtained closer to the point of progression to cancer. Another notable finding was the lack of difference in macrophage distributions between lesions with high and low methylation levels, possibly indicating that the observed patterns reflect intrinsic immune dysfunction rather than lesion severity. This discrepancy may be explained by the timing of these events: shifts in macrophage polarization likely occur closer to malignant transformation, while elevated methylation levels can arise much earlier. Additionally, macrophage densities had higher variability than methylation levels.

Although the present study cannot establish mechanistic causality, several features of the spatial and phenotypic data suggest plausible drivers of macrophage polarization during progression to cancer. Lesions that progressed showed a similar overall T-cell infiltration but relatively lower expression of activation markers on T-cells, indicating the emergence of a functionally immunoregulatory or exhausted state that may influence macrophage polarization. Within this context, M2-like macrophages were preferentially located at the epithelial-stromal interface and co-localized with FOXP3^+^ T-cells. This preferential co-localization in progressive HSIL is consistent with a self-reinforcing immunosuppressive niche described in other tumor types: M2-polarized macrophages secrete CCL22 and TGF-β, which recruit and sustain Tregs, while Tregs in turn produce IL-10 and IL-4 that further stabilize M2 polarization. In premalignant cervical and head-and-neck lesions, this macrophage-Treg axis has been associated with immune evasion and resistance to immune-mediated clearance.^26^ By contrast, regressing lesions displayed more frequent interactions between M1-like macrophages and CD8^+^ T-cells, which indicates a more effective local effector response. Notably, the most pronounced polarization patterns were observed in lesions sampled closest to cancer diagnosis, in contrast to methylation, which is high across all progressive samples, suggesting that progressing HSIL actively remodels the surrounding microenvironment. This temporal dissociation suggests that epigenetic and immune microenvironmental changes follow distinct kinetics during anal carcinogenesis. DNA methylation is an early, cumulative event in HPV-driven carcinogenesis that is already established at the precancerous stage,^27^ whereas our longitudinal data indicate that immune remodeling intensifies in a narrow window proximal to malignant transformation. This is consistent with findings in lung, breast, and oral premalignancies, where the transition to invasive disease is accompanied by a late-stage switch toward immunosuppressive myeloid phenotypes.^28^^,^^29^ Together, these observations support a model in which immune evasion, adaptive T-cell dysfunction, and stromal immunosuppression act together to drive macrophage polarization in HSIL destined to progress, whereas their absence or attenuation characterizes lesions that regress.

### Limitations of the study

First, the number of regressive and progressive HSIL samples was small, owing to their rarity; the achieved power at a large effect size (0.8) was 0.80 (Kruskal-Wallis) and 0.05 (Mann-Whitney *U*). Consequently, findings from the IMC cohort are preliminary and hypothesis-generating and require independent validation in larger series. This limitation reflects the exceptional rarity of outcome-defined anal HSIL specimens. To mitigate this, we incorporated an independent cross-sectional HSIL cohort using surrogates of cancer risk (HPV status and morphology, methylation levels, and nadir CD4 cell counts). However, because of the variability, many observed trends still did not reach statistical significance. Second, two HIV-negative individuals were included among the progressive HSIL cases due to limited sample availability; a sensitivity analysis excluding these participants shows similar results. As the cohort is predominantly composed of people living with HIV, these findings apply primarily to this population and should be generalized to HIV-negative individuals with caution. Third, reliance on archival specimens resulted in incomplete clinical metadata, impeding the inclusion of all cases in every analysis.

**Table 1:** Participant characteristics of included individuals in the imaging mass cytometry and immunofluorescence analysis

	Imaging Mass Cytometry								Immunofluorescence									
	No dysplasia		Regressive HSIL		Progressive HSIL		Anal cancer		No dysplasia		Regressive HSIL		Progressive HSIL		Anal cancer		Cross-sectional HSIL	
Patients (*N*)	1	–	10	–	6	–	3	–	9	–	5	–	14	–	13	–	62	–
Biopsies (*N*)	1	–	10	–	9	–	3	–	9	–	5	–	26	–	13	–	62	–
Age (Median, IQR)	62	N/A	53.5	47–59.75	58.5	52.75–61.25	51	49–56.5	51	44–54	51	50–61	58.5	51.5–61.5	57	51–59	48	41–54
Gender (*N*, % participants)																		
W	0	0%	0	0%	0	0%	0	0%	0	0%	0	0%	2	14%	1	8%	0	0%
M	1	100%	10	100%	6	100%	3	100%	9	100%	5	100%	12	86%	12	92%	61	98%
TW	0	0%	0	0%	0	0%	0	0%	0	0%	0	0%	0	0%	0	0%	1	2%
HIV-status (*N*, % participants)																		
HIV+	1	100%	10	100%	6	100%	3	100%	9	100%	5	100%	10	71%	11	85%	62	100%
HIV−	0	0%	0	0%	0	0%	0	0%	0	0%	0	0%	4	29%	2	15%	0	0%
Nadir CD4 cell count (*N*, % participants)																		
<200 cells/μL	0	0%	0	0%	0	0%	0	0%	0	0%	0	0%	0	0%	0	0%	16	26%
>200 cells/μL	0	0%	0	0%	0	0%	0	0%	0	0%	0	0%	0	0%	0	0%	12	19%
Unknown/N/A	1	100%	10	100%	6	100%	3	100%	9	100%	5	100%	14	100%	13	100%	34	55%
AIN grade (*N*, % biopsies)																		
AIN2	N/A	N/A	8	80%	3	33%	N/A	N/A	N/A	N/A	4	80%	8	31%	N/A	N/A	36	58%
AIN3	N/A	N/A	2	20%	6	67%	N/A	N/A	N/A	N/A	1	20%	18	69%	N/A	N/A	26	42%
HPV status (*N*, % biopsies)																		
Pos	0	0%	9	90%	9	100%	3	100%	1	11%	4	80%	22	85%	12	92%	62	100%
Neg	1	100%	1	10%	0	0%	0	0%	7	78%	0	0%	0	0%	0	0%	0	0%
Unknown	0	0%	0	0%	0	0%	0	0%	1	11%	1	20%	4	15%	1	8%	0	0%
HPV 16(*N*, % biopsies)																		
Pos	0	0%	4	40%	8	89%	3	100%	0	0%	2	40%	19	73%	10	77%	28	45%
Neg	1	100%	6	60%	1	11%	0	0%	8	89%	2	40%	3	12%	2	15%	29	47%
Unknown	0	0%	0	0%	0	0%	0	0%	1	11%	1	20%	4	15%	1	8%	5	8%
Combined (*N*, % biopsies)																		
HPV16+AIN3	N/A	N/A	1	10%	6	67%	N/A	N/A	N/A	N/A	1	20%	14	54%	N/A	N/A	12	19%
HPV16-AIN2	N/A	N/A	6	60%	2	22%	N/A	N/A	N/A	N/A	2	40%	3	12%	N/A	N/A	16	26%
other	N/A	N/A	3	30%	1	11%	N/A	N/A	N/A	N/A	1	20%	5	19%	N/A	N/A	29	47%
Unknown	N/A	N/A	0	0%	0	0%	N/A	N/A	N/A	N/A	1	20%	4	15%	N/A	N/A	5	8%
Methylation (*N*, % biopsies)																		
high	0	0%	0	0%	7	78%	3	100%	0	0%	0	0%	26	100%	13	100%	22	35%
intermediate	0	0%	7	70%	2	22%	0	0%	0	0%	3	60%	0	0%	0	0%	19	31%
low	1	100%	3	30%	0	0%	0	0%	9	100%	2	40%	0	0%	0	0%	21	34%
Unknown	0	0%	0	0%	0	0%	0	0%	0	0%	0	0%	0	0%	0	0%	0	0%

HSIL, high-grade squamous intraepithelial lesions; HPV, human papillomavirus; AIN, anal intraepithelial neoplasia.

## Resource availability

### Lead contact

Requests for further information and resources should be directed to and will be fulfilled by the lead contact, Henry J.C. de Vries (h.j.devries@amsterdamumc.nl).

### Materials availability

This study did not generate new unique reagents.

### Data and code availability

• The data supporting this study are pseudonymized and cannot be made openly available due to privacy regulations (GDPR/UAVG) and the scope of the original informed consent. Data are available from the lead contact upon reasonable request, subject to institutional approval and a data transfer agreement.
• All original code has been deposited at Zenodo and is publicly available at Zenodo: https://doi.org/10.5281/zenodo.21862047 as of the date of publication.
• Any additional information required to reanalyze the data reported in this paper is available from the lead contact upon request subject to institutional approval and a data transfer agreement. Please find an overview of resources used and accession codes in the key resources table.

## Acknowledgments

We thank Gabrielle Krebbers, Sylvia Duin, and Timo ter Braak for excellent technical assistance. We thank Nikki Thuijs for her assistance with the histopathological assessment. We thank Shiva Najjary and Rehana Hewavisenti for their assistance with the interpretation of the results. This work was supported by the 10.13039/100006753AMC PhD Scholarship program 2020. The sources of funding did not have any influence on the design of the study and will have no influence on collection, analysis, interpretation of the data, in writing the manuscript, and in the decision to submit the article for publication.

## Author contributions

F.D.G.L., J.M.P., R.D.M.S., and H.J.C.d.V. were involved in conception and design of the study. F.D.G.L. and H.J.C.d.V. wrote the manuscript. M.E.I. and N.F.C.C.d.M. performed the IMC analysis. C.J.M.v.N. performed the histopathological assessment. M.E.I., J.F.V., and N.F.C.C.d.M. performed the immunofluorescence analysis. K.R. provided statistical expertise. All authors critically reviewed the manuscript and approved the final version.

## Declaration of interests

R.D.M.S. is minority stockholder of Self-screen B.V., a spin-off company of VUmc, which owns patents on methylation markers and HPV detection. H.J.C.d.V. received financial compensation or goods for research from Medigene, Gilead, and MSD; financial compensation for presentations from Abbott and Janssen; and financial compensation for advice to Medigene and Novartis.

## STAR★Methods

### Key resources table

**Table:** 

REAGENT or RESOURCE	SOURCE	IDENTIFIER
**Antibodies**		

Anti-human CD8a (clone D8A8Y)	CST	CAT# 85336BF
Anti-human PD-1 (clone D4W2J)	CST	CAT# 86163BF
Anti-human ICOS (clone D1K2T(tm))	CST	CAT# 89601BF
Anti-human CD204 (clone J5HTR3)	Thermo Fisher	CAT# 14-9054-95; RRID:AB_2865330
Anti-human CD103 (clone EPR4166(2))	Abcam	CAT# ab221210
Anti-human Tbet (clone 4B10)	Biolegend	CAT# 644825; RRID:AB_2563788
Anti-human CD19 (clone D4V4B)	CST	CAT# 9664BF
Anti-human CD163 (clone D6U1J)	CST	CAT# 93498BF
Anti-human TGFbeta (clone TB21)	Thermo Fisher	CAT# MA5-16949; RRID:AB_2538424
Anti-human HLA-DR (clone TAL 1B5)	Abcam	CAT# ab176408; RRID:AB_3492081
Anti-human CD11b (clone D6X1N)	CST	CAT# 49420BF
Anti-human Granzyme B (clone D6E9W)	CST	CAT# 46890BF
Anti-human cleaved caspase (clone 5A1E)	CST	CAT# 15372BF
Anti-human CD39 (clone EPR20627)	Abcam	CAT# ab236038; RRID:AB_2943150
Anti-human VISTA (clone D1L2G(TM))	CST	CAT# 64953BF
Anti-human CD14 (clone D7A2T)	CST	CAT# 56082BF
Anti-human CD56 (clone E7X9M)	CST	CAT# 99746BF
Anti-human CD7 (clone EPR4242)	Abcam	CAT# ab230834; RRID:AB_2889384
Anti-human CD11c (clone EP1347Y)	Abcam	CAT# ab216655; RRID:AB_2864379
Anti-human TCRgd +2ND Ab (clone H41)	Santa Cruz	CAT# sc-100289; RRID:AB_1130061
Anti-human CD4 +2ND AB (clone EPR6855)	Abcam	CAT# ab181724; RRID:AB_2864377
Anti-human CD45 (clone D9M8I)	CST	CAT# 13917BF
Anti-human CD3 (clone EP449E)	Abcam	CAT# ab271850; RRID:AB_3698033
Anti-human PD-L1 (clone E1L3N(R))	CST	CAT# 13684BF
Anti-human FOXP3 (clone D608R)	CST	CAT# 12653BF
Anti-human CD27 (clone EPR8569)	Abcam	CAT# 92803BF
Anti-human Vimentin (clone D21H3)	CST	CAT# 5741BF
Anti-human Keratin (clone C11 and AE1/AE3)	Biolegend	CAT# 4545BF
Anti-human B catenin (clone D10A8)	CST	CAT# 8480BF
Anti-human CD20 (clone H1)	CST	CAT# 48750BF
Anti-human CD68 (clone D4B9C)	CST	CAT# 76437BF
Anti-human CD31 (clone 89C2)	CST	CAT# 3528BF
Anti-human CD57 (clone HNK-1/Leu-7)	Abcam	CAT# ab269781
Anti-human Ki-67 (clone 8D5)	CST	CAT# 9449BF
Anti-human P16ink4a (clone D3W8G)	CST	CAT# 45208BF
Anti-human IDO (clone D5J4E(TM))	CST	CAT# 86630BF
Anti-human CD45RO (clone UCHL1)	CST	CAT# 55618BF
Anti-human D2-40 (clone D2-40)	Biolegend	CAT# 916606; RRID:AB_2565820
Anti-human CD38 (clone EPR4106)	Abcam	CAT# ab226034; RRID:AB_3719518
Anti-human CD15 (clone MC480)	CST	CAT# 4744BF
Anti-human Histone H3 (clone D1H2)	CST	CAT# 4499BF

**Critical commercial assays**		

*ASCL1* and *ZNF582* methylation assay: PreCursor-M AnoGYN Methylation Assay (RUO)	Self-Screen	CAT# QHR16100
Opal 6-Plex Manual Detection Kit	Akoya	Part# NEL811001KT

**Software and algorithms**		

MCD viewer	Standard BioTools	Software - Standard BioTools
CyTOF	Standard BioTools	Software - Standard BioTools
InForm	Quanterix, Akoya	inForm: Automated Tissue Analysis for Multimarker Studies | Quanterix
QuPath	QuPath Github open software	QuPath
R version 4.3.2	R-project open software	R: The R Project for Statistical Computing
R code	Newly created. Deposited at Zenodo	Zenodo: https://doi.org/10.5281/zenodo.21862047

### Experimental model and study participant details

This study consisted of two subsequent phases aimed at characterizing and validating immune-microenvironment markers in anal HSIL.

In the characterization phase, imaging mass cytometry using CYTOF was used to identify immunological differences associated with HSIL progression by comparing four anal disease categories: one non-dysplastic control biopsy, 10 biopsies from 10 patients with HSIL lesions that underwent complete, spontaneous regression within one year (regressive HSIL), 9 biopsies from 6 patients with HSIL lesions that progressed to invasive anal squamous cell carcinoma (SCC) over time (progressive HSIL), and 3 SCC specimens. Regressive HSIL was defined as a histologically confirmed HSIL lesion followed by at least two consecutive biopsies without HSIL at the same anatomic site within 12 months, in the absence of ablative treatment. Progressive HSIL was defined as a histologically confirmed HSIL lesion in a patient who subsequently developed invasive SCC at the same anatomic location. Longitudinal biopsies were available for the progressive HSIL group because patients were under active surveillance with periodic high-resolution anoscopy (HRA). Anatomic concordance of serial biopsies was ensured by clinical mapping during HRA, with lesion location documented by clock-face position and distance from the anal verge at each visit. Specimens without dysplasia and those with regressive HSIL, collected between 2015 and 2023, were retrieved from the Amsterdam University Medical Centers (Amsterdam UMC) pathology archive, as previously described.^30^^,^^31^ Progressive HSIL specimens and SCC specimens, collected between 2000 and 2023, were obtained from the Amsterdam UMC pathology archive as well as from residual material of a previously studied cohort from the archives of Amsterdam UMC and five other hospitals in the Netherlands.^30^^,^^31^^,^^32^

In the validation phase, identified immunological differences were examined by multispectral immunofluorescence across three cohorts: (1) A replication cohort mirroring the discovery set to confirm differential expression: 9 non-dysplastic biopsies, 5 regressive HSIL biopsies, 14 progressive HSIL biopsies from a longitudinal cohort of 14 patients (the biopsy closest to progression to cancer), and 13 SCC biopsies. (2) A total of 26 biopsies from the longitudinal cohort of the 14 patients with serial HSIL biopsies collected at one or more time points prior to progression to cancer, allowing temporal analysis of marker dynamics.(3) A larger, cross-sectional HSIL series, consisting of 62 biopsies, used to relate marker expression to surrogate indicators of anal cancer risk: high versus low DNA methylation levels (*N* = 43), HPV16^+^HSIL-AIN3 versus HPV^−^HSIL-AIN2 (*N* = 57), and nadir CD4 count (e.g., lowest CD4 value in lifetime) of <200 cells/μL versus ≥200 cells/μL (*N* = 28). This series was obtained from leftover material of a prior cohort of men who have sex with men and transgender women living with HIV, collected between 2008 and 2017.^32^ All participants were living with HIV, except for 4 in the progressive HSIL group and 2 in the SCC group of the validation cohort (Table 1).

We adhered to the Declaration of Helsinki and the Code of Conduct for Responsible Use of Leftover Material of the Dutch Federation of Biomedical Scientific Societies. All participants gave written informed consent. Ethical approval was waived by the Institutional Review Board of the Amsterdam UMC, location Academic Medical Center, under reference numbers 18/316 (regressive HSIL and non-dysplastic control biopsies), 17/234 (progressive HSIL biopsies), 18/333 (SCC biopsies), and 07/318 (cross-sectional HSIL biopsies). For the progressive HSIL series, local ethical approval was granted by the NHS Health Research Authority, United Kingdom (IRAS ID 226196), and the Ethical Committee of the University Witten/Herdecke, Germany (reference no. 166/2017).

### Method details

Demographic and clinical data, including age, sex, gender, and HIV status were extracted from electronic patient records. During routine screening visits, patients underwent a digital anorectal examination and high-resolution anoscopy (HRA) following current guidelines.^33^^,^^34^ Biopsies were obtained when abnormalities were detected on inspection. Next, biopsies were formalin-fixed and paraffin-embedded (FFPE). Histopathological review, HPV genotyping, and DNA methylation analysis were performed as previously described.^30^^,^^32^ A board-certified pathologist (C. J. M. v. N.) confirmed the histopathological classification of all samples, with p16^INK4A^ immunohistochemistry applied as indicated. Bisulfite-converted DNA was then analyzed for six methylation markers using two multiplex quantitative methylation-specific PCR (qMSP) assays, each targeting three genes along with the reference gene, β-actin (ACTB). A logistic model from a methylation marker panel with the genes *ASCL1* and *ZNF582* was used to calculate predicted probabilities.^30^ Samples with predicted probabilities below the first quartile of the original validation set were classified as low-methylation, whereas those above the third quartile were classified as high-methylation. Samples between the first and third quartile were classified as intermediate-methylation.

#### Imaging mass cytometry

Four-micrometer tissue sections were mounted on silane-coated glass slides, dried overnight at 37°C, and stored at 4°C until use. Carrier-free IgG antibodies were conjugated to purified lanthanide metals using the MaxPar antibody labeling kit (Standard BioTools). Following the protocol of Ijsselsteijn et al.,^35^ tissue sections underwent deparaffinization (three 5-min immersions in xylene) and rehydration through graded ethanol solutions. Antigen retrieval was performed using a diluted 10× low pH buffer preheated in a microwave, followed by boiling and cooling to room temperature. Sections were blocked with Superblock buffer (Thermo Fisher Scientific) and incubated with primary antibodies in two steps—a 5-h incubation at room temperature and an overnight incubation at 4°C. Following antibody staining, sections were washed, incubated with Intercalator Ir (1.25 μM, Standard BioTools), and then air-dried prior to ablation. Regions of interest (ROIs) (1000 × 1000 μm) were selected based on hematoxylin and eosin staining of consecutive sections, ensuring full tissue representation. The Hyperion imaging mass cytometry system (Standard BioTools) was calibrated using a 3-element tuning slide, with a threshold set at 1500 mean duals detected for 175Lu. Antibody information is shown in Table S1.

Imaging mass cytometry (IMC) data was first visually expected in the MCD viewer^tm^ (Standard BioTools) and images with poor quality or folded tissue were excluded. Sample normalization, cell segmentation and phenotype identification were done as described previously.^36^

Phenotype counts from the CYTOF analysis were visualized using clustered heatmaps and jitter plots with crossbars displaying individual data points. Visual comparisons were performed between regressive and progressive HSIL samples, and any observed differences were further examined using immunofluorescence. Neighborhood enrichments between pairs of cellular phenotypes were visualized by a heatmap. For the heatmaps, mean cell counts per phenotype (normalized to tissue area, cells/mm^2^) and methylation levels were calculated for each sample. These values were then standardized (*Z* score) across samples for each phenotype independently, centering and scaling so that the resulting values represent relative deviations from the cohort mean. To define cellular neighbourhoods, for each cell the direct neighbors in a 20 μm radius were identified. To define specific interactions, compared to random occurrences of cells due to high cell numbers, a 1000 itterations premutation test was used.

#### Immunofluorescence

Multiplex immunofluorescence staining was performed on FFPE HSIL tissue slides using primary antibodies against CD68, CD163, CD204, HLADR, and pan-cytokeratin. Tissue slides were deparaffinized, rehydrated, and subjected to heat-induced epitope retrieval in citrate buffer. After blocking with Superblock solution, slides were incubated with primary antibodies, followed by Poly-HRP secondary antibody and Opal reagents for signal amplification. Each staining round was followed by antibody stripping in citrate buffer to allow sequential staining of multiple markers. DAPI was used as a nuclear counterstain, and slides were mounted with Prolong Gold Antifade Reagent. Imaging was conducted using the Vectra 3.0 Automated Quantitative Pathology Imaging System at 20× magnification, capturing multiple snapshots per tissue biopsy. Antibody information is shown in Table S2.

Spectral unmixing of Opal dyes was performed using InForm software, and image analysis, including cell segmentation and phenotype training, was conducted using QuPath, with nuclei segmented based on DAPI staining and cells contoured accordingly. Phenotype training involved manually selecting approximately 20 cells per phenotype across 30 biopsy images.

Samples without dysplasia, regressive HSIL, progressive HSIL, and SCC were compared with each other. Additionally, the progression timeline from HSIL to cancer was analyzed. Finally, the cross-sectional HSIL series was evaluated with methylation status, morphology and HPV status, and nadir CD4 cell count serving as surrogate markers for cancer progression risk.^32^^,^^37^^,^^38^^,^^39^ This allowed us to study the phenotype profiles in relation to these major risk factors. Specifically, HSIL samples with high methylation (predicted probability above the third quartile) were compared with those having low methylation (predicted probability below the first quartile), HPV16-positive HSIL-AIN3 samples were compared with HPV16-negative HSIL-AIN2 samples, and subjects with a low nadir CD4 cell count (<200 cells/μL) with subjects with a high nadir CD4 cell count (>200 cells/μL). To focus on the extremes, samples with intermediate methylation (predicted probability between first and third quartile), as well as those with HPV16-positive AIN2 or HPV16-negative AIN3, were excluded from the analysis. Additionally, cross-sectional HSIL lesions were stratified by AIN grade and plotted together with control and cancer samples to show correlations with morphology.

### Quantification and statistical analysis

The distribution of macrophage phenotypes was visualized using jitter plots with crossbars, with separate analyses performed for absolute cell counts (normalized by tissue area) and percentages of total CD68^+^ macrophages. To compare differences across groups, the Kruskal-Wallis test was used, and the Mann-Whitney U test for intergroup comparisons. To account for multiple comparisons in pairwise group analyses, *p*-values derived from Mann-Whitney U tests were adjusted using the Bonferroni correction. For each cell phenotype, all pairwise comparisons between the four study groups (Control, Reg HSIL, Prog HSIL, and SCC) were considered simultaneously, yielding six possible pairs per phenotype. The Bonferroni-corrected *p*-values (mw_p_adj) were used to determine statistical significance, with a threshold of *p* < 0.05. This was performed for the main comparison as well as for the cross-sectional HSIL cohort. To assess whether HIV status biased macrophage-phenotype comparisons, we performed a sensitivity analysis repeating all pairwise Mann-Whitney U tests after excluding subjects without HIV and compared Bonferroni-adjusted *p*-values with the full-cohort analysis. To evaluate the relative macrophage enrichment, we derived the cell density ratio of “M2-like” (CD163^+^CD204^+/−^HLA-DR^-^ and CD163^+/−^CD204^+^HLA-DR^-^) versus “M1-like” (CD163^+/−^CD204^+/−^HLA-DR^+^) macrophages, and compared these ratios across clinical groups with jitter plots (median ± IQR) and pairwise Mann-Whitney U tests.

For participants with HSIL collected at one or more time points prior to progression to cancer, we conducted longitudinal analyses to track how the targeted immune-cell populations changed over time. To assess temporal changes in macrophage subsets, linear regression models were fitted for each phenotype using time before SCC as a continuous predictor. Separate models were constructed for both absolute cell counts, percentages of total CD68^+^ macrophages, and the “M2-like” vs. “M1-like” ratio.

Sample size calculations were performed using the Kruskal-Wallis test with Mann-Whitney U post-hoc pairwise comparisons (4 groups, one-sided, α = 0.05, power = 0.80). The estimated required sample sizes per group were 637, 104, and 42 for small (f = 0.2), medium (f = 0.5), and large (f = 0.8) effect sizes, respectively. As the present study relied on availability of rare samples, the sample size was below these estimates.

R version 4.3.2 was used with packages: “circlize”, “ComplexHeatmap”, “dplyr”, “ggplot2”, “ggpubr”, “lemon”, “purr”, “scales”, “stringr”, “tidyr”, “viridis”.
