# Supplementary material for: Distinct tumor microenvironment profiles characterize regressive versus progressive anal precancers
Source: iScience. 2026 Sep 21;29(10):117404. doi: 10.1016/j.isci.2026.117404 (PMC13626763; doi:10.1016/j.isci.2026.117404)
Supplement: Supplementary file 1 [file mmc1.pdf]

iScience, Volume *29*

Supplemental information

Distinct tumor microenvironment profiles characterize regressive versus progressive anal precancers

Fernando Dias Goncalves Lima, Marieke E. Ijsselsteijn, Johanna F. Verkerk, Kirsten Rozemeijer, Carel J.M. van Noesel, Noel F.C.C. de Miranda, Jan M. Prins, Rosalie M. Luiten, Renske D.M. Steenbergen, and Henry J.C. de Vries

Supplementary File

Supplementary Figures

S1 Macrophage counts imaging mass cytometry

Macrophage densities across anal disease categories measured by imaging mass cytometry. Each point shows area-normalized cell densities (cells per mm^2) for the different macrophage phenotypes within an individual biopsy. Point colors represent the disease categories (Control: grey, Regressive HSIL: green, Progressive HSIL: orange, and SCC: black). Cross-bars mark the median, with the tops and bottoms of the bars corresponding to the first and third quartiles, respectively.

Abbreviations: HSIL: high-grade anal squamous intraepithelial lesions; SCC: squamous cell carcinoma.

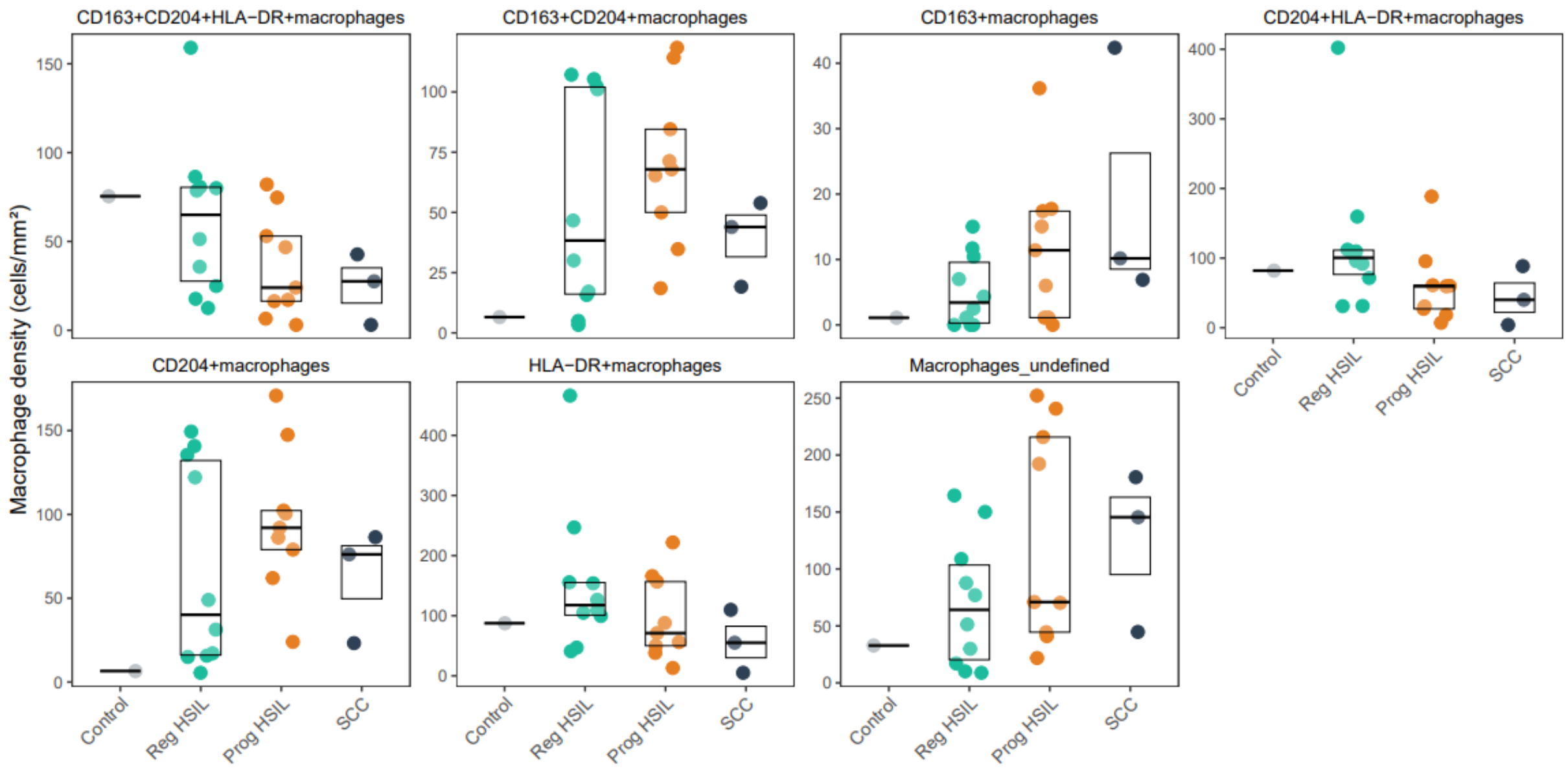

S2 Macrophage proportions imaging mass cytometry

Macrophage densities across anal disease categories measured by imaging mass cytometry. Each point shows the percentage of total macrophages attributable to a given phenotype within an individual biopsy. Point colors represent the disease categories (Control: grey, Regressive HSIL: green, Progressive HSIL: orange, and SCC: black). Cross-bars mark the median, with the tops and bottoms of the bars corresponding to the first and third quartiles, respectively.

Abbreviations: HSIL: high-grade anal squamous intraepithelial lesions; Prog HSIL: progressive HSIL; Reg HSIL: regressive HSIL SCC: squamous cell carcinoma.

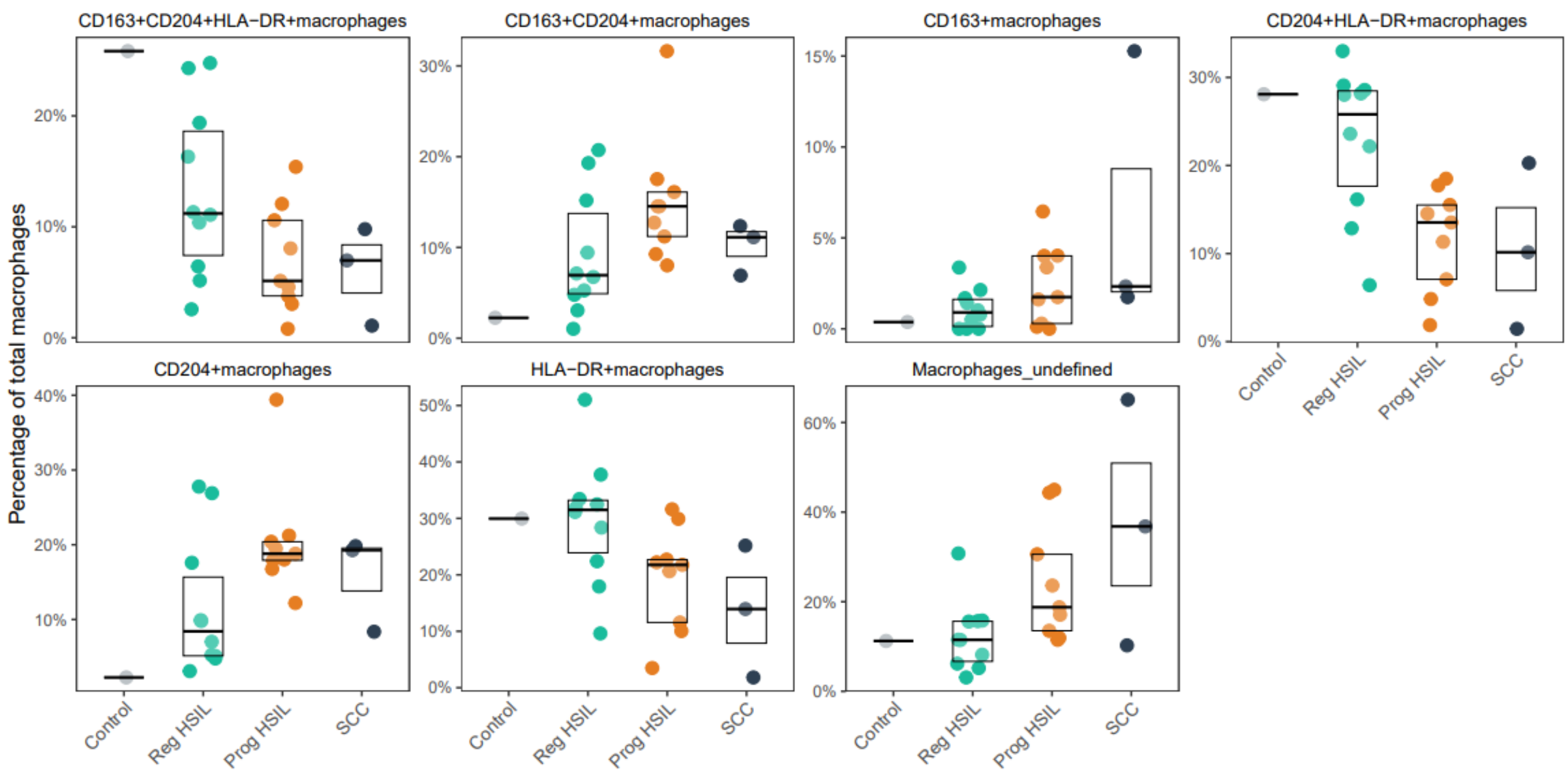

S3 Expression of activation markers in T-cells

T-cell densities across anal disease categories measured by imaging mass cytometry. Each point shows the percentage of total T-cells attributable to a given phenotype within an individual biopsy. Point colors represent the disease categories (Control: black, Regressive HSIL: green, Progressive HSIL: red, and SCC: grey). Horizontal bars mark the median.

Abbreviations: HSIL: high-grade anal squamous intraepithelial lesions; Progressive: progressive HSIL; Regressive: regressive HSIL SCC: squamous cell carcinoma.

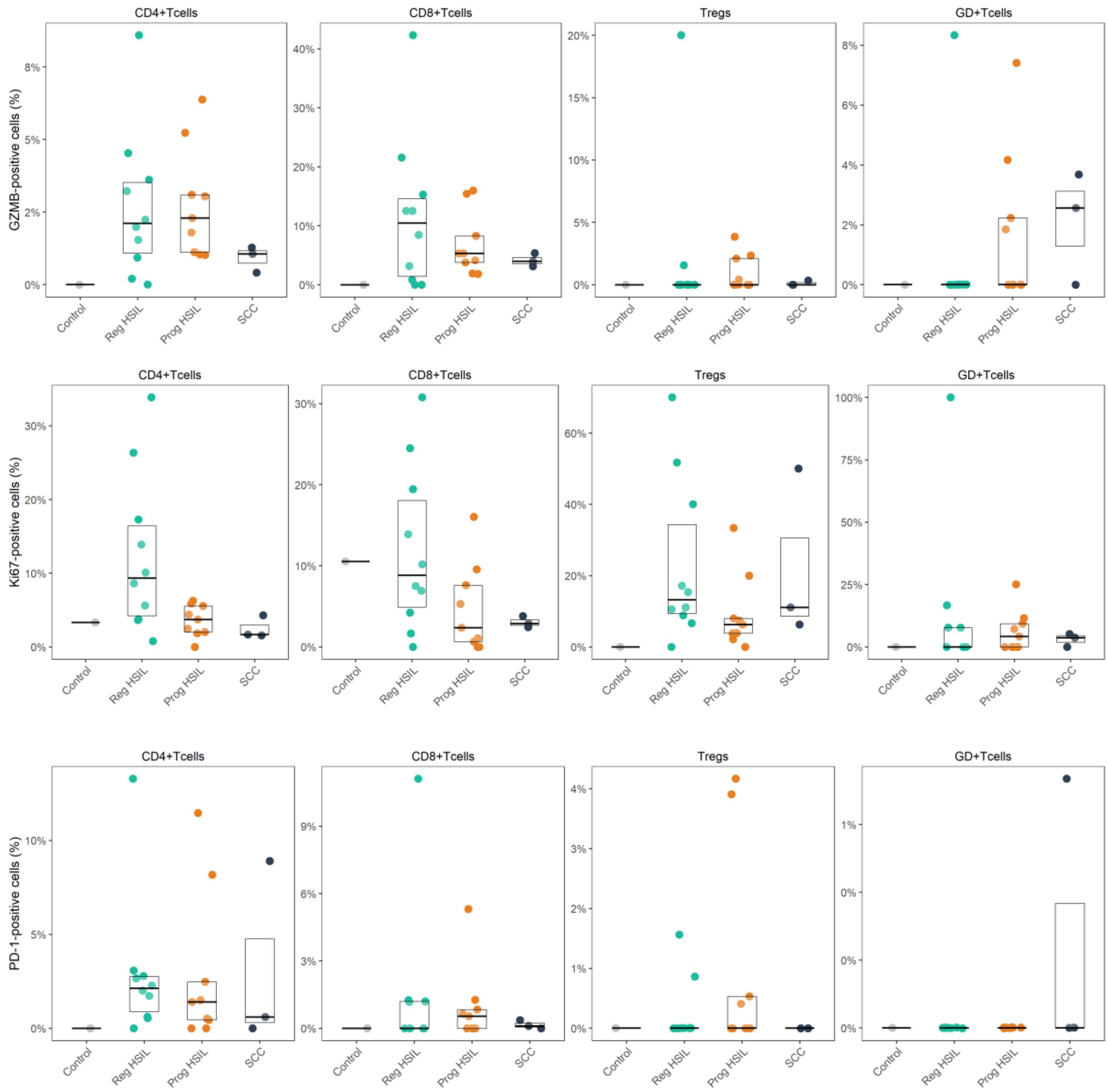

S4 Macrophage proportions immunofluorescence

Proportion of the macrophage phenotypes across anal disease categories measured by immunofluorescence. Each point shows the percentage of total macrophages attributable to a given phenotype within an individual biopsy. Point colors represent the disease categories (Control: grey, Regressive HSIL: green, Progressive HSIL: orange, and SCC: black). Cross-bars mark the median, with the tops and bottoms of the bars corresponding to the first and third quartiles, respectively.

*=p< 0.05; **=p<0.01.

Abbreviations: HSIL: high-grade anal squamous intraepithelial lesions; Prog HSIL: progressive HSIL; Reg HSIL: regressive HSIL SCC: squamous cell carcinoma.

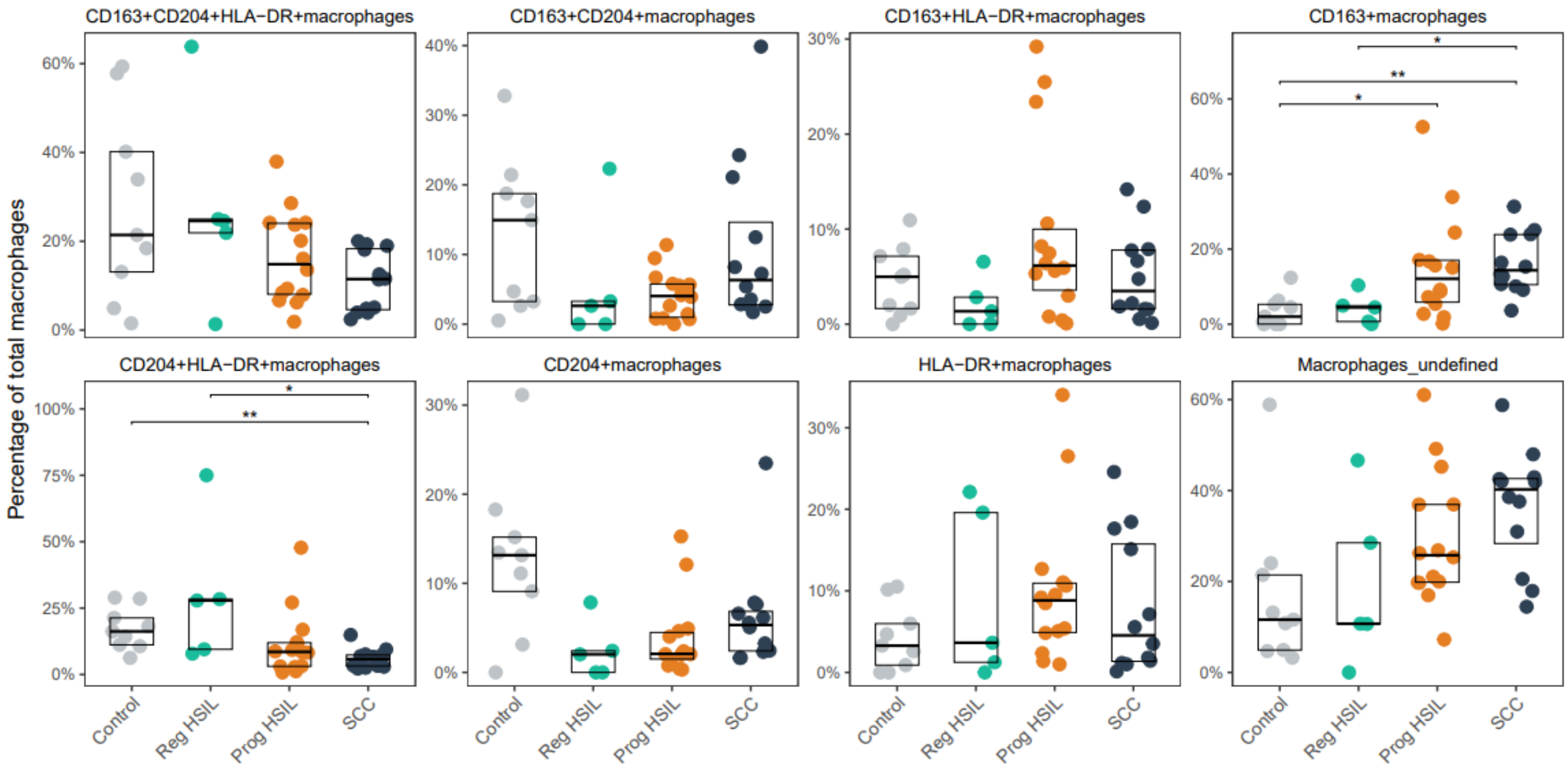

S5 Sensitivity analysis on inclusion of HIV negative subjects for macrophage proportions immunofluorescence

Proportion of the macrophage phenotypes across anal disease categories measured by immunofluorescence, excluding subjects without HIV. Each point shows the percentage of total macrophages attributable to a given phenotype within an individual biopsy. Point colors represent the disease categories (Control: grey, Regressive HSIL: green, Progressive HSIL: orange, and SCC: black). Cross-bars mark the median, with the tops and bottoms of the bars corresponding to the first and third quartiles, respectively. The table below show the comparisons whose adjusted p-value crossed the 0.05 threshold after excluding subjects without.

*=*p*< 0.05; **=*p*<0.01.

Abbreviations: HSIL: high-grade anal squamous intraepithelial lesions; Prog HSIL: progressive HSIL; Reg HSIL: regressive HSIL SCC: squamous cell carcinoma.

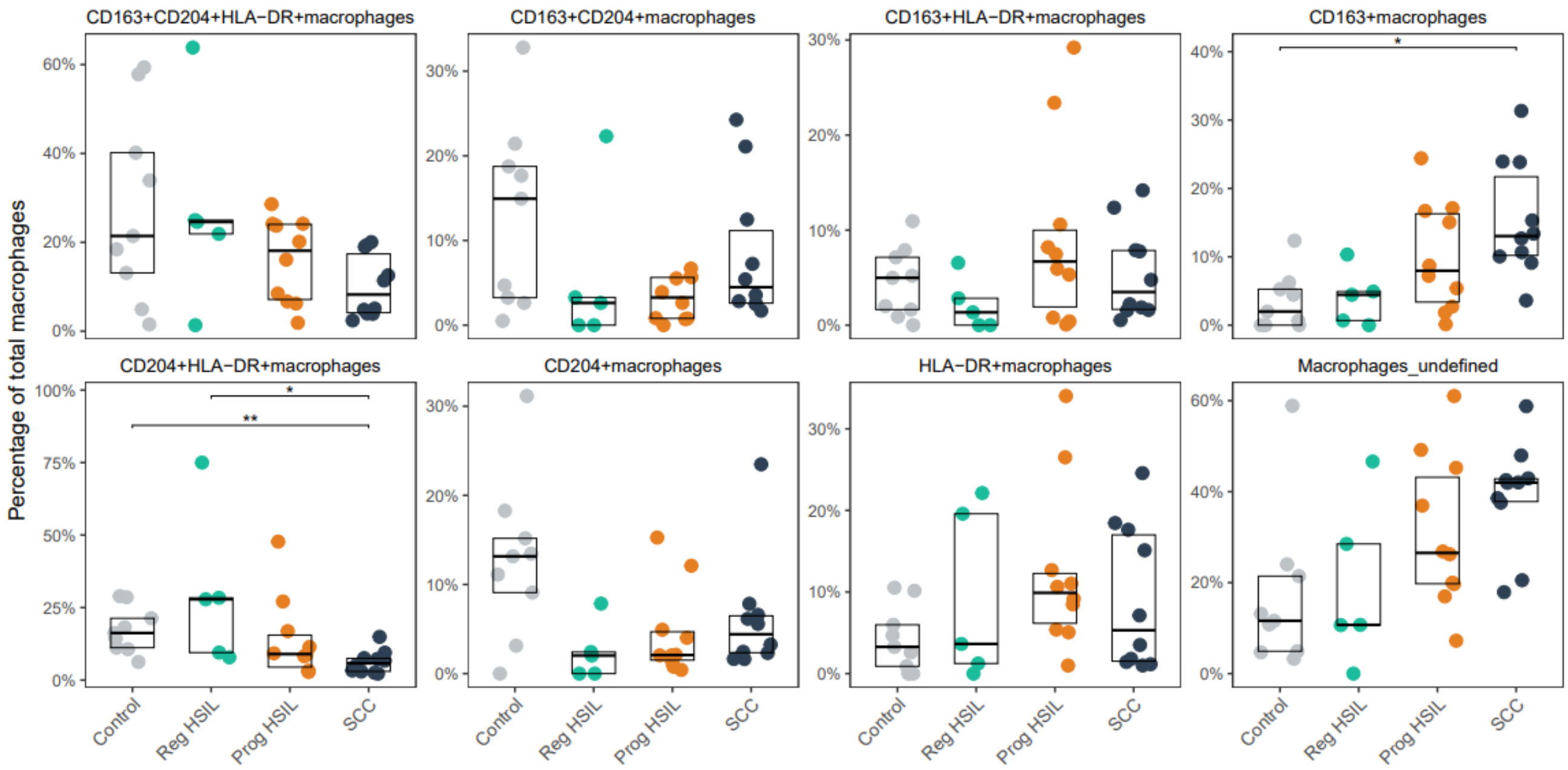

Comparisons whose adjusted p-value crossed the 0.05 threshold after excluding subjects without:

phenotype	group1	group2	p-value	Adjusted p-value after exclusion subjects without HIV
1 CD163macrophages	ND	prog	0.044	0.222
2 CD163macrophages	reg	SCC	0.037	0.076

S6 – M2-like/M1-like ratio

For each biopsy, the densities of M2-like macrophages, defined as $CD163^{+}CD204^{+/-}HLA\text{-}DR^{-}$ and $CD163^{+/-}CD204^{+}HLA\text{-}DR^{-}$, and M1-like macrophages, defined as $CD163^{+/-}CD204^{+/-}HLA\text{-}DR^{+}$ (cells/mm^{2}) were calculated, and their ratio (M2-like/(M1-like+M2-like)) was plotted. Point colors represent the disease categories (Control: grey, Regressive HSIL: green, Progressive HSIL: orange, and SCC: black). Cross-bars mark the median, with the tops and bottoms of the bars corresponding to the first and third quartiles, respectively.

Abbreviations: HSIL: high-grade anal squamous intraepithelial lesions; IMC: imaging mass cytometry; Prog HSIL: progressive HSIL; Reg HSIL: regressive HSIL SCC: squamous cell carcinoma.

IMC

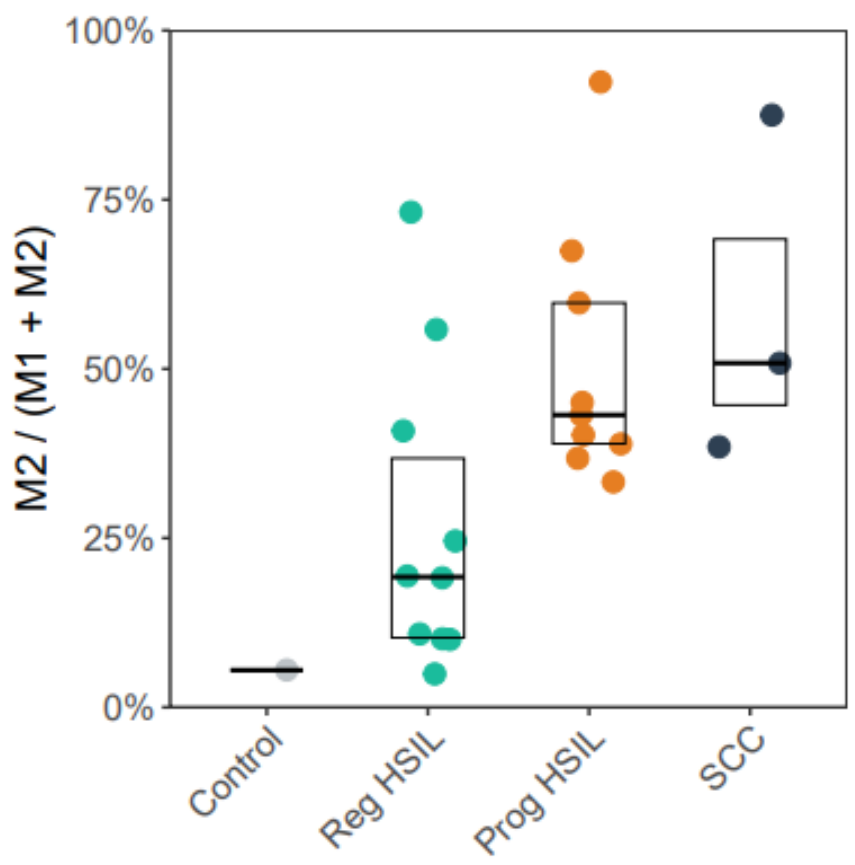

Immunofluorescence

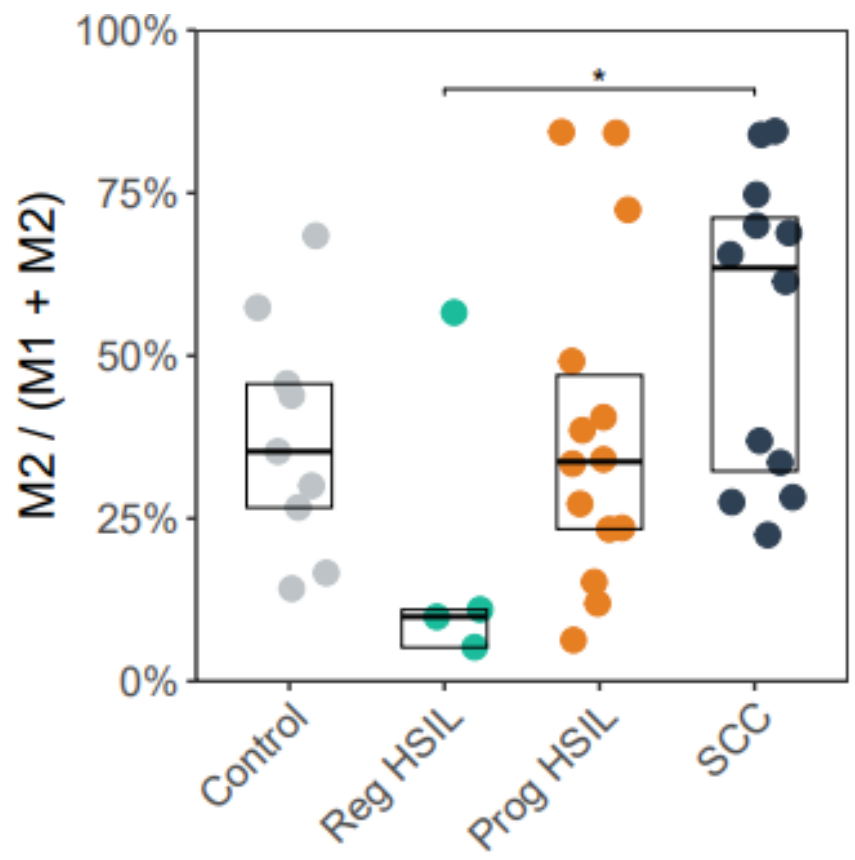

S7 – Stromal macrophage counts immunofluorescence

Macrophage densities in the stromal compartments across anal disease categories measured by immunofluorescence. Each point shows area-normalized cell densities (cells per mm²) for the different macrophage phenotypes within an individual biopsy. Point colors represent the disease categories (Control: grey, Regressive HSIL: green, Progressive HSIL: orange, and SCC: black). Cross-bars mark the median, with the tops and bottoms of the bars corresponding to the first and third quartiles, respectively.

*=*p*< 0.05; **=*p*<0.01; ***=p<0.001.

Abbreviations: HSIL: high-grade anal squamous intraepithelial lesions; Prog HSIL: progressive HSIL; Reg HSIL: regressive HSIL SCC: squamous cell carcinoma.

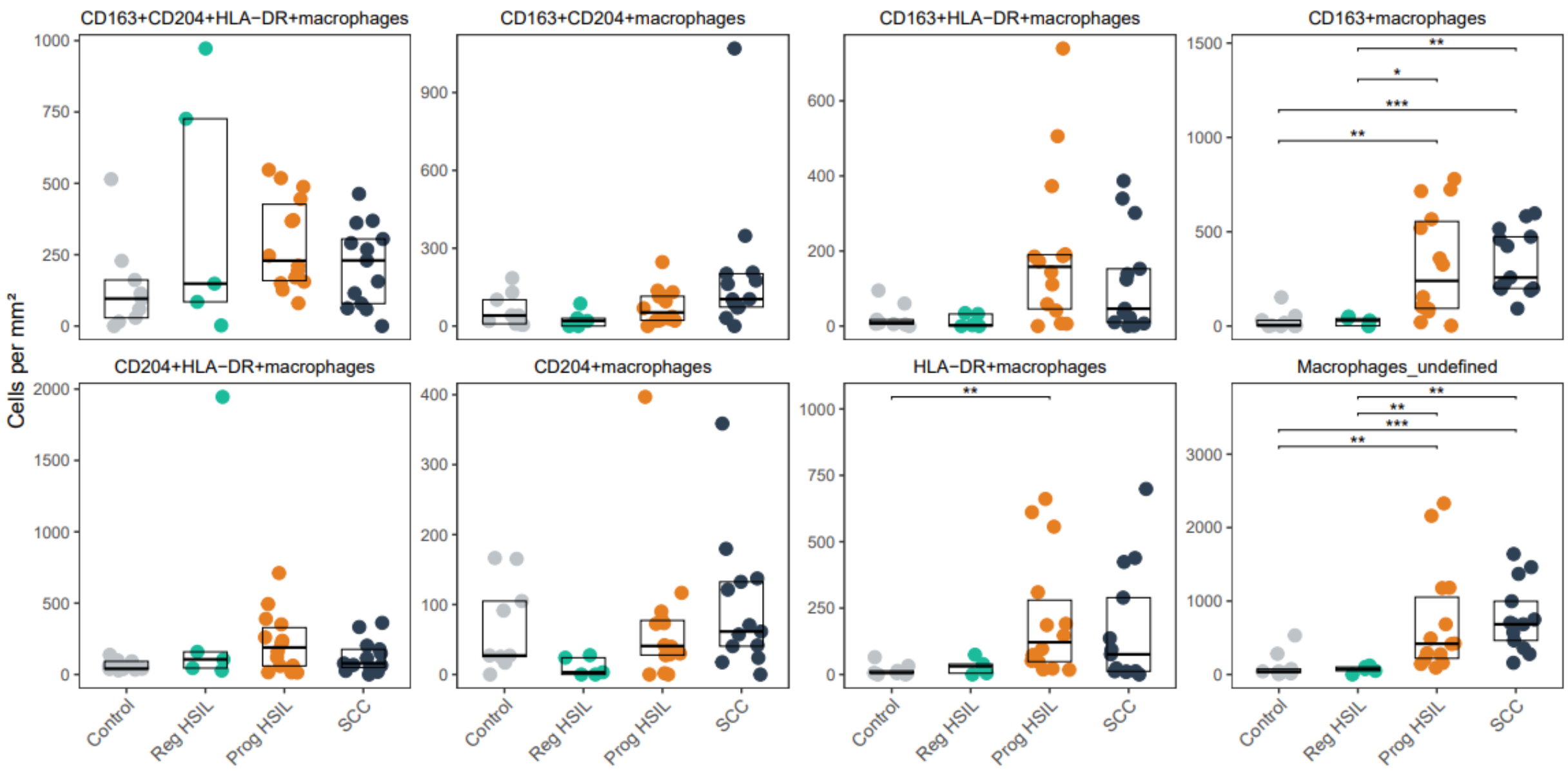

S8 – Epithelial macrophage counts immunofluorescence

Macrophage densities in the epithelial compartments across anal disease categories measured by immunofluorescence. Each point shows area-normalized cell densities (cells per mm^2) for the different macrophage phenotypes within an individual biopsy. Point colors represent the disease categories (Control: grey, Regressive HSIL: green, Progressive HSIL: orange, and SCC: black). Cross-bars mark the median, with the tops and bottoms of the bars corresponding to the first and third quartiles, respectively.

*=*p*< 0.05; **=*p*<0.01; ***=p<0.001.

Abbreviations: HSIL: high-grade anal squamous intraepithelial lesions; Prog HSIL: progressive HSIL; Reg HSIL: regressive HSIL SCC: squamous cell carcinoma.

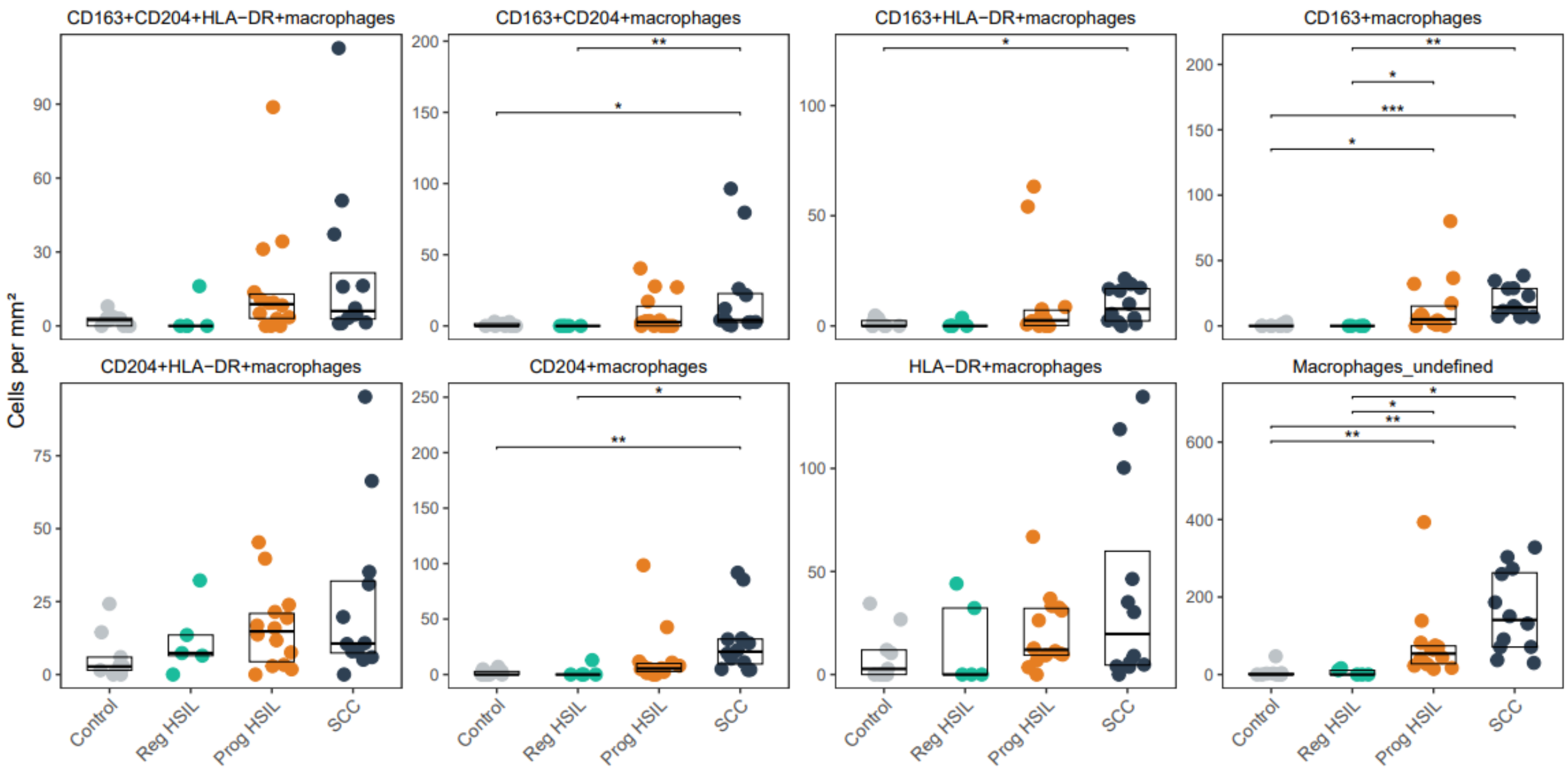

S9 – Temporal stromal macrophage counts in HSIL progressing to cancer

Temporal dynamics of macrophage subpopulations in the stromal compartments during progression from HSIL to SCC. Scatter plots show area-normalized cell densities (cells per mm^2) for different macrophage phenotypes as a function of time before SCC diagnosis; points at time = 0 correspond to SCC biopsies. Dashed lines represent the fitted linear regression, and grey shaded bands the 95 % confidence intervals.

Abbreviations: ns: non-significant.

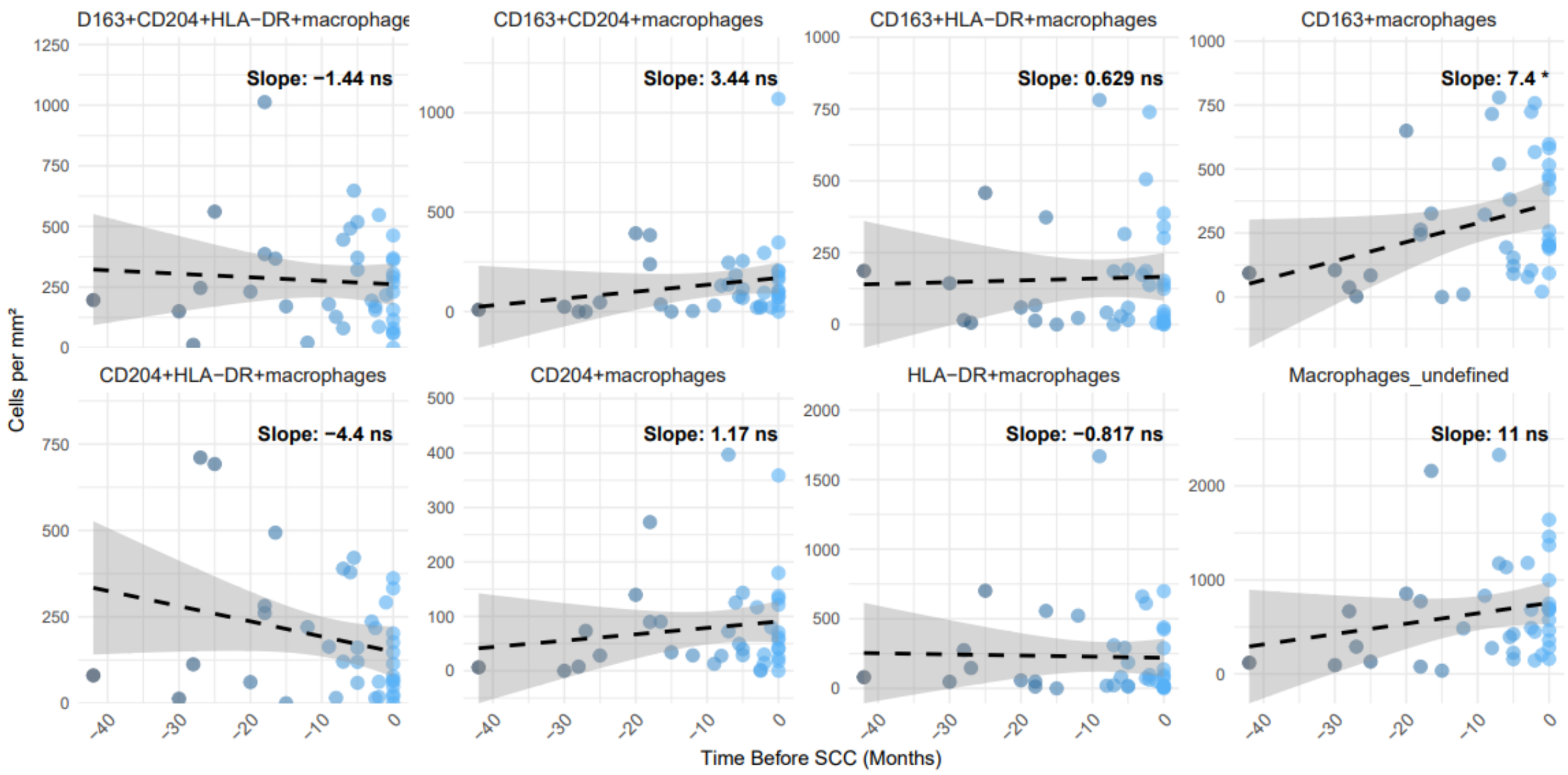

S10 – Temporal epithelial macrophage counts in HSIL progressing to cancer

Temporal dynamics of macrophage subpopulations in epithelial compartments during progression from HSIL to SCC. Scatter plots show area-normalized cell densities (cells per mm^2) for different macrophage phenotypes as a function of time before SCC diagnosis; points at time = 0 correspond to SCC biopsies. Dashed lines represent the fitted linear regression, and grey shaded bands the 95 % confidence intervals.

Abbreviations: ns: non-significant.

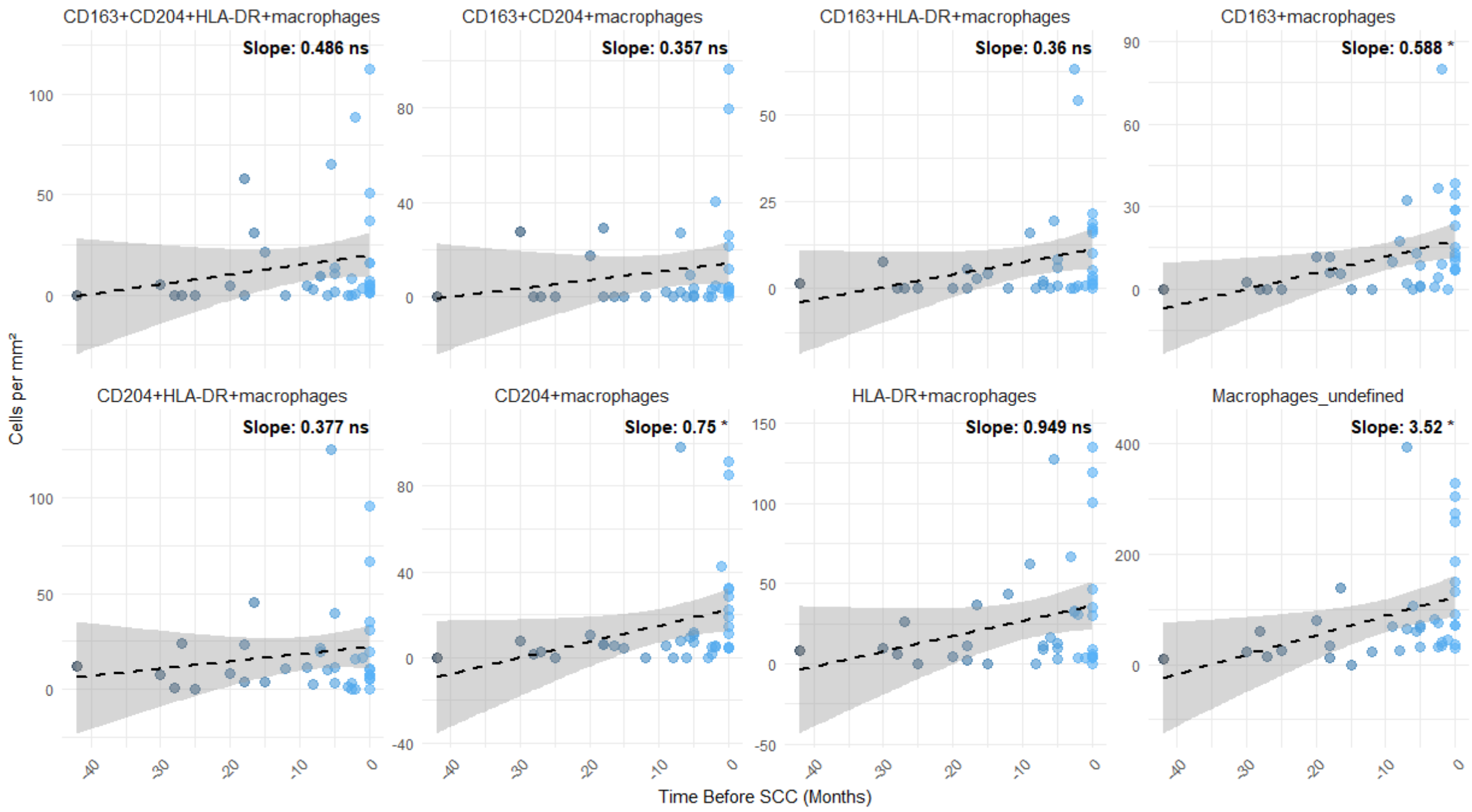

S11 – Temporal stromal macrophage proportions in HSIL progressing to cancer

Temporal dynamics of macrophage subpopulations in the stromal compartments during progression from HSIL to SCC. Scatter plots show the percentage of total macrophages attributable to a given phenotype as a function of time before SCC diagnosis; points at time = 0 correspond to SCC biopsies. Dashed lines represent the fitted linear regression, and grey shaded bands the 95 % confidence intervals.

Abbreviations: ns: non-significant.

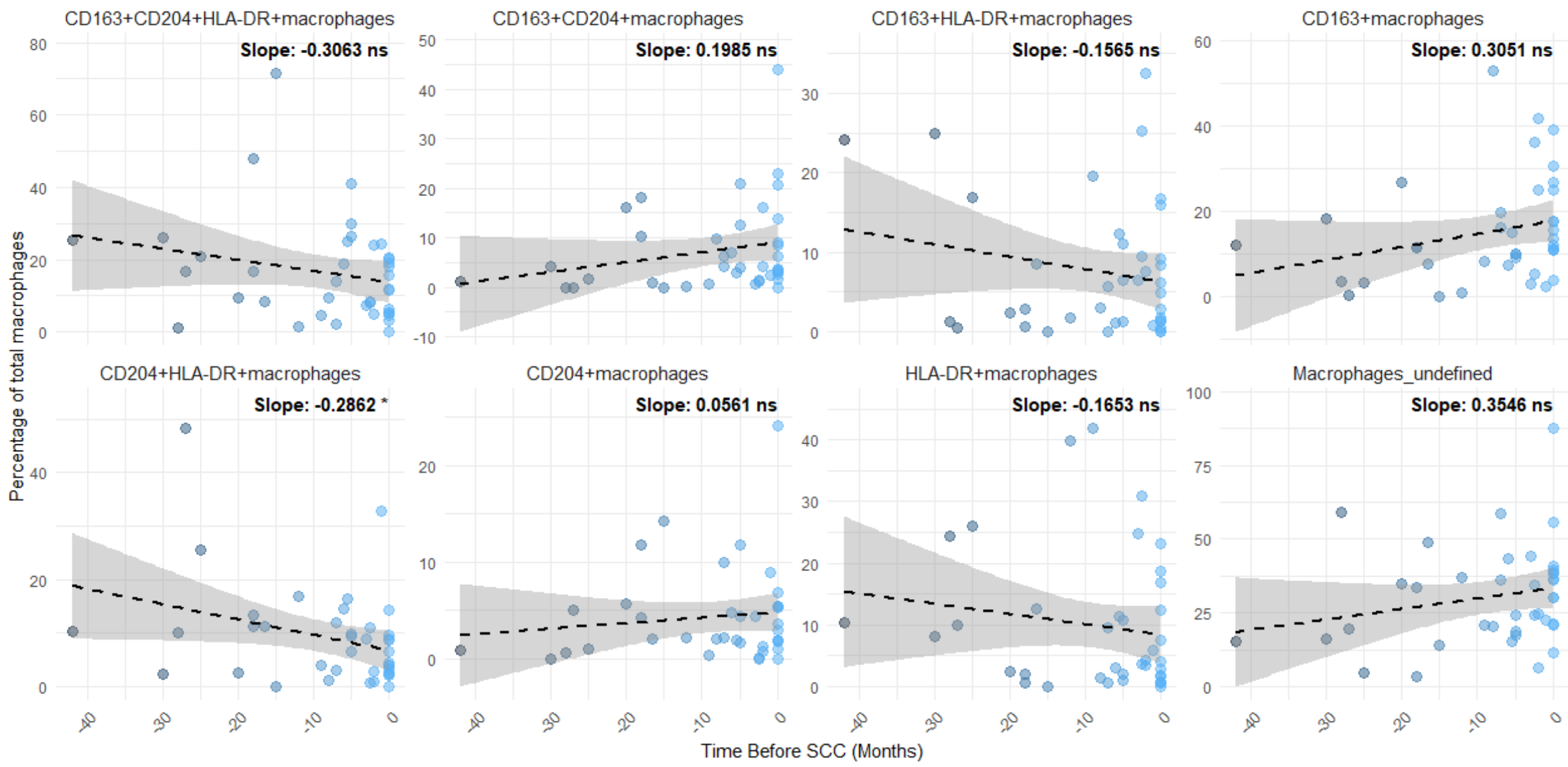

S12 – Temporal epithelial macrophage proportions in HSIL progressing to cancer

Temporal dynamics of macrophage subpopulations in the epithelial compartments during progression from HSIL to SCC. Scatter plots show the percentage of total macrophages attributable to a given phenotype as a function of time before SCC diagnosis; points at time = 0 correspond to SCC biopsies. Dashed lines represent the fitted linear regression, and grey shaded bands the 95 % confidence intervals.

Abbreviations: ns: non-significant.

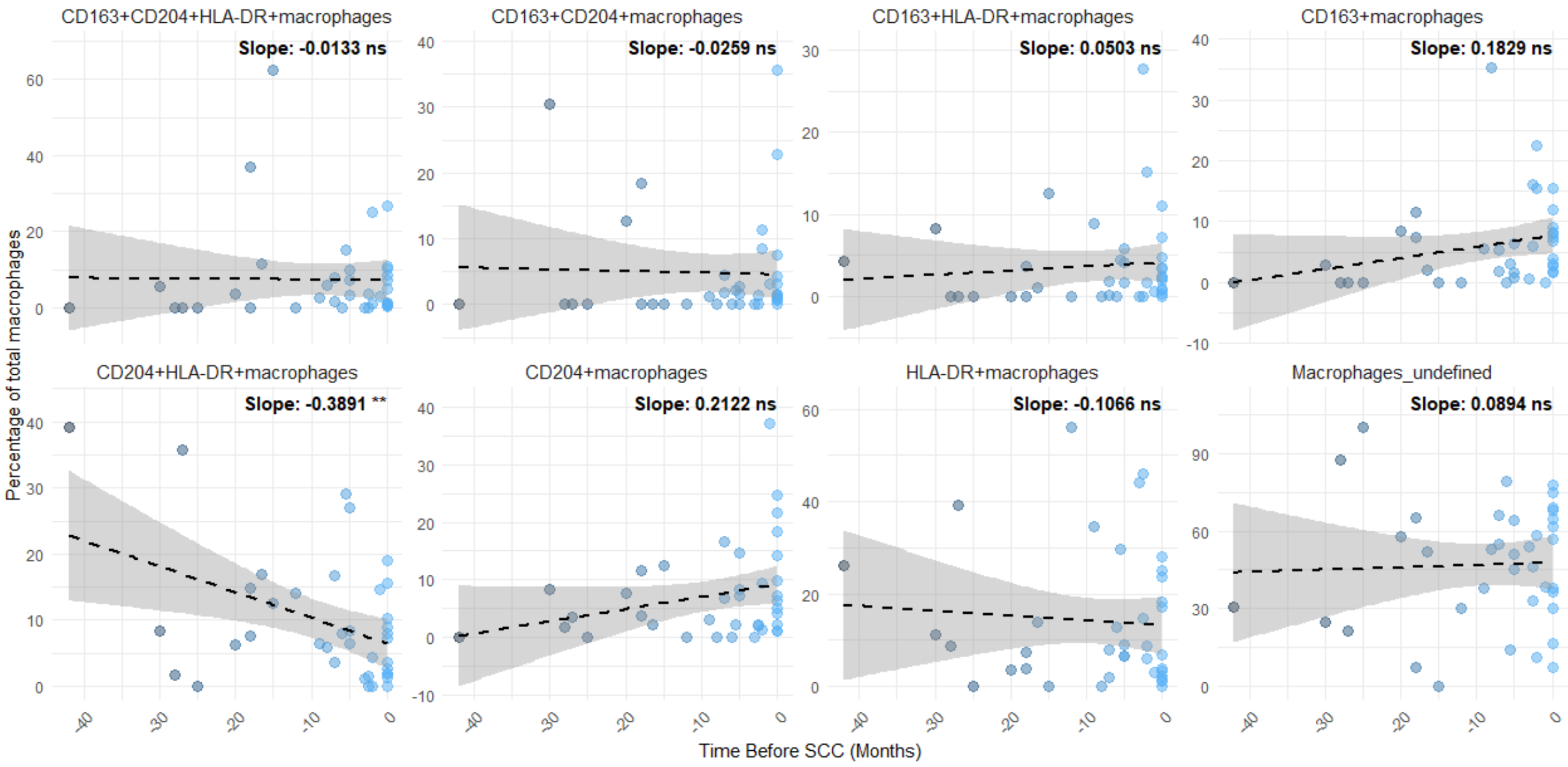

S13 – Temporal macrophage proportions in HSIL progressing to cancer, M2-like/M1-like ratio

Temporal dynamics of macrophage subpopulations in the epithelial compartments during progression from HSIL to SCC. For each biopsy, the densities of M2-like macrophages, defined as $CD163^{+}CD204^{+/-}HLA\text{-}DR^{-}$ and $CD163^{+/-}CD204^{+}HLA\text{-}DR^{-}$, and M1-like macrophages, defined as $CD163^{+/-}CD204^{+/-}HLA\text{-}DR^{+}$ (cells/mm²) were calculated, and their ratio (M2-like/(M1-like+M2-like)) was plotted. Scatter plots show the M2-like/M1-like ratio as a function of time before SCC diagnosis; points at time = 0 correspond to SCC biopsies. Dashed lines represent the fitted linear regression, and grey shaded bands the 95 % confidence intervals.

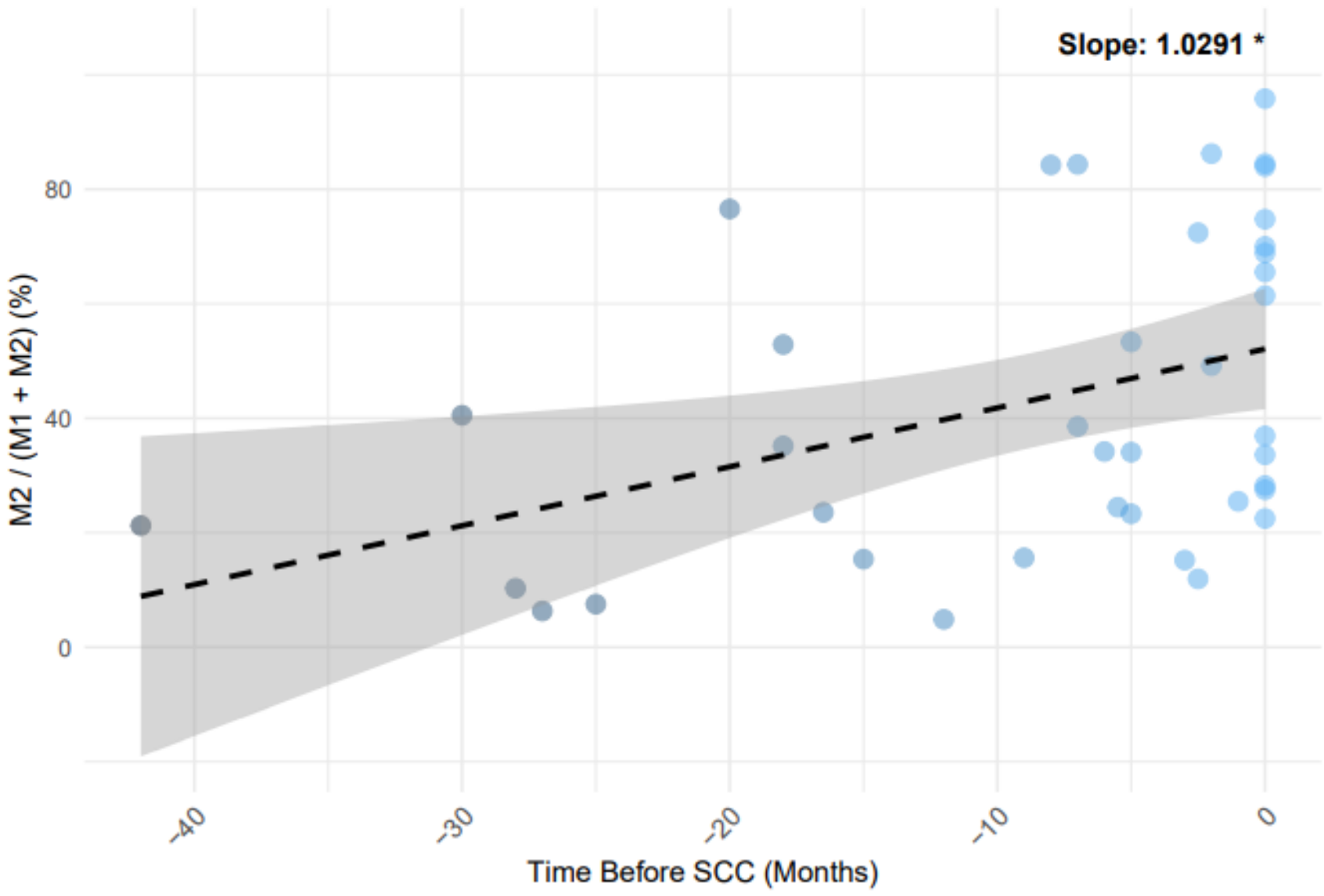

S14 – Temporal dynamics of DNA methylation levels in HSIL progressing to cancer

Temporal dynamics of DNA methylation levels during progression from HSIL to SCC. For each biopsy, the predicted methylation probability was derived from a DNA methylation-based classifier containing the markers *ASCL1* and *ZNF582*. Scatter plots show the methylation level as a function of time before SCC diagnosis; points at time = 0 correspond to SCC biopsies. Dashed lines represent the fitted linear regression, and grey shaded bands the 95% confidence intervals.

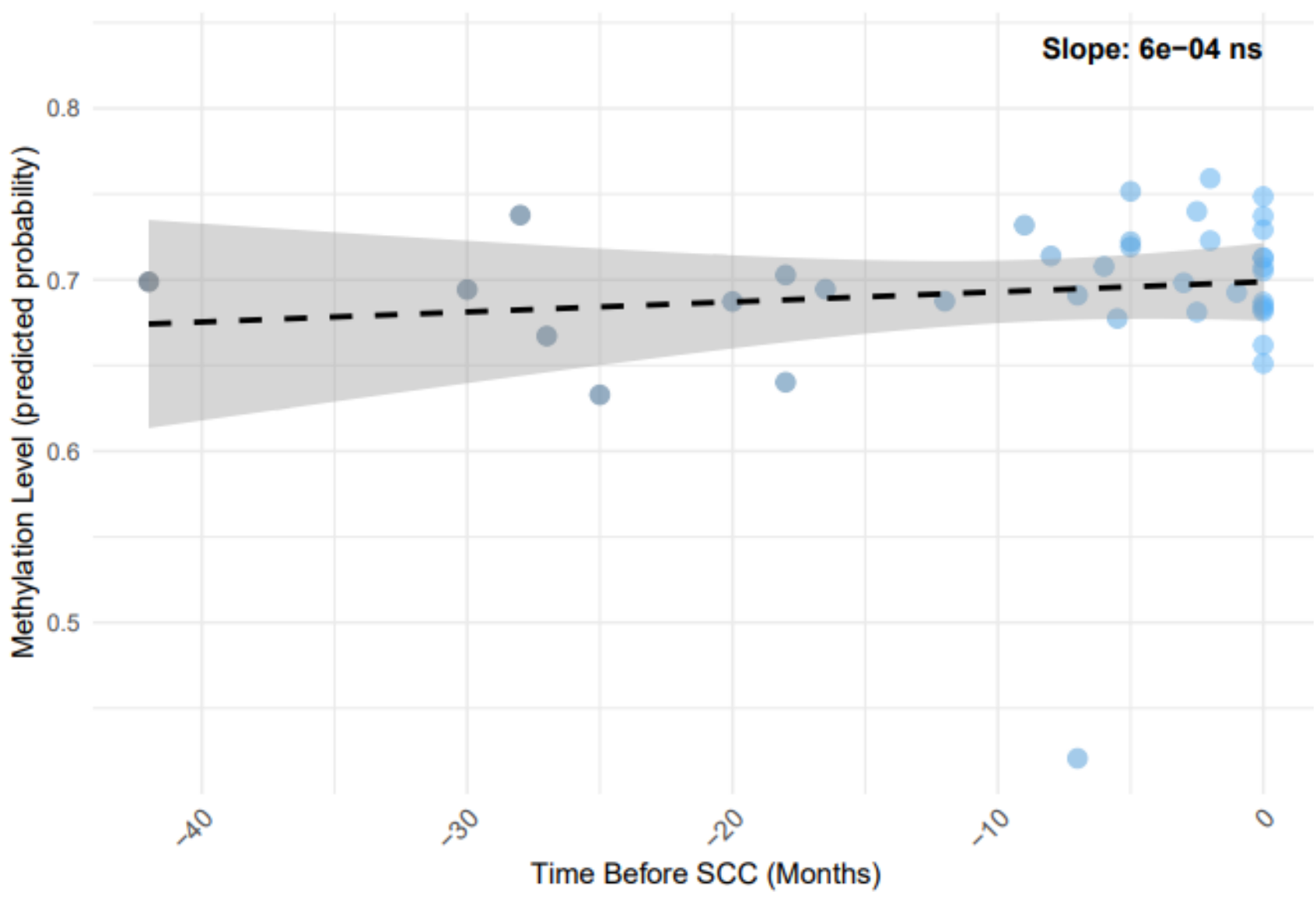

S15 – Temporal dynamics of macrophage proportions and DNA methylation heatmap

Longitudinal dynamics of macrophage balance and DNA methylation levels during progression from HSIL to SCC. Each row represents one patient; columns represent sequential biopsies aligned by time relative to SCC diagnosis, with pre-SCC biopsies right-aligned (oldest to most recent, left to right) and the SCC biopsy shown in the rightmost column. Grey cells indicate absent biopsies at that timepoint. Numbers within cells indicate the time in months before SCC diagnosis; "SCC" denotes the diagnostic biopsy (time = 0). Upper panel: M2-like/M1-like macrophage balance, expressed as the percentage of M2-like macrophages (CD163+CD204+/−HLA-DR− and CD163+/−CD204+HLA-DR−) relative to the total M1+M2-like population (cells/mm²). Lower panel: Predicted DNA methylation probability derived from a DNA methylation-based classifier. In both panels, blue indicates low values, white intermediate values, and red-orange high values.

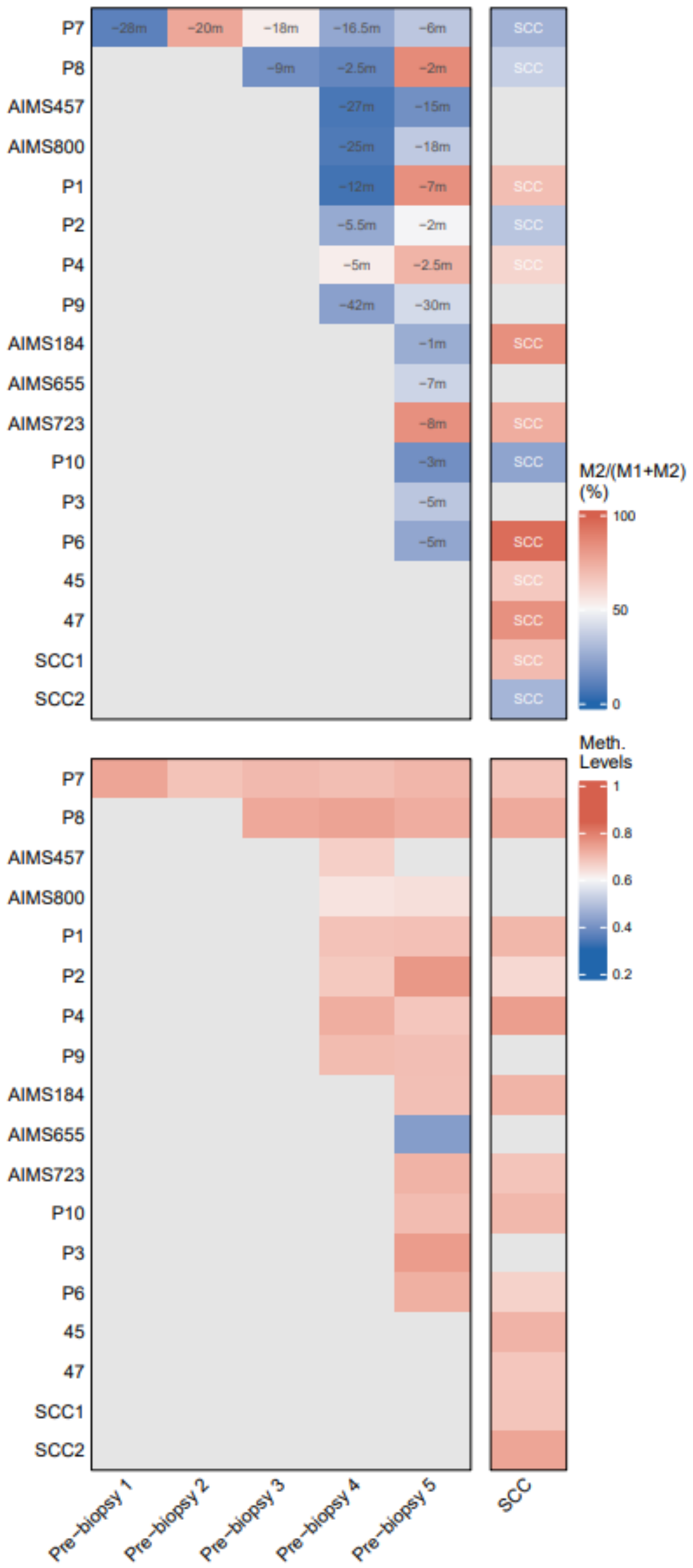

S16 – Stromal macrophage counts in HSIL stratified by methylation status, HPV+AIN3, and nadir CD4 cell count

Macrophage densities in stromal compartments of cross-sectional anal HSIL samples measured by immunofluorescence. Each point shows area-normalized cell densities (cells per mm^2) for the different macrophage phenotypes within an individual biopsy. Samples are stratified by three patient-level factors: DNA methylation status (low *vs* high), histopathological grade combined with HPV genotype (HPV16$^+$AIN3 *vs* HPV16$^-$AIN2) and nadir CD4$^+$T-cell count (<200 *vs* ≥200 cells/µl). Point colors represent the different patient-level factors. Cross-bars mark the median, with the tops and bottoms of the bars corresponding to the first and third quartiles, respectively.

Abbreviations: AIN: anal intraepithelial neoplasia grade; HSIL: high-grade anal squamous intraepithelial lesions; SCC: squamous cell carcinoma.

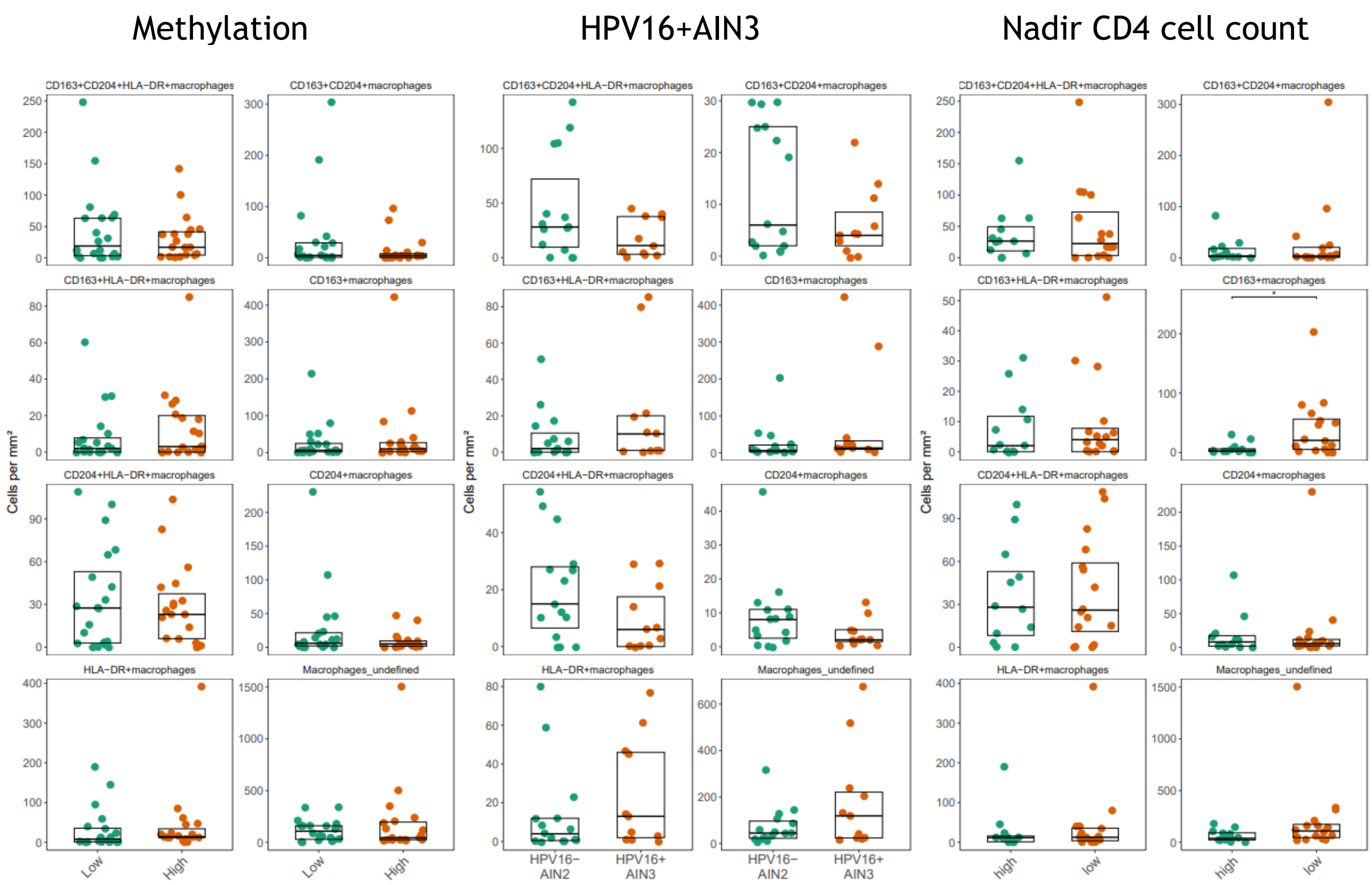

S17 – Epithelial macrophage counts in HSIL stratified by methylation status, HPV+AIN3, and nadir CD4 cell count

Macrophage densities in epithelial compartments of cross-sectional anal HSIL samples measured by immunofluorescence. Each point shows area-normalized cell densities (cells per mm^2) for the different macrophage phenotypes within an individual biopsy. Samples are stratified by three patient-level factors: DNA methylation status (low *vs* high), histopathological grade combined with HPV genotype (HPV16^{+}AIN3 *vs* HPV16^{-}AIN2) and nadir CD4^{+}T-cell count (<200 *vs* ≥200 cells/µl). Point colors represent the different patient-level factors. Cross-bars mark the median, with the tops and bottoms of the bars corresponding to the first and third quartiles, respectively.

Abbreviations: AIN: anal intraepithelial neoplasia grade; HSIL: high-grade anal squamous intraepithelial lesions; SCC: squamous cell carcinoma.

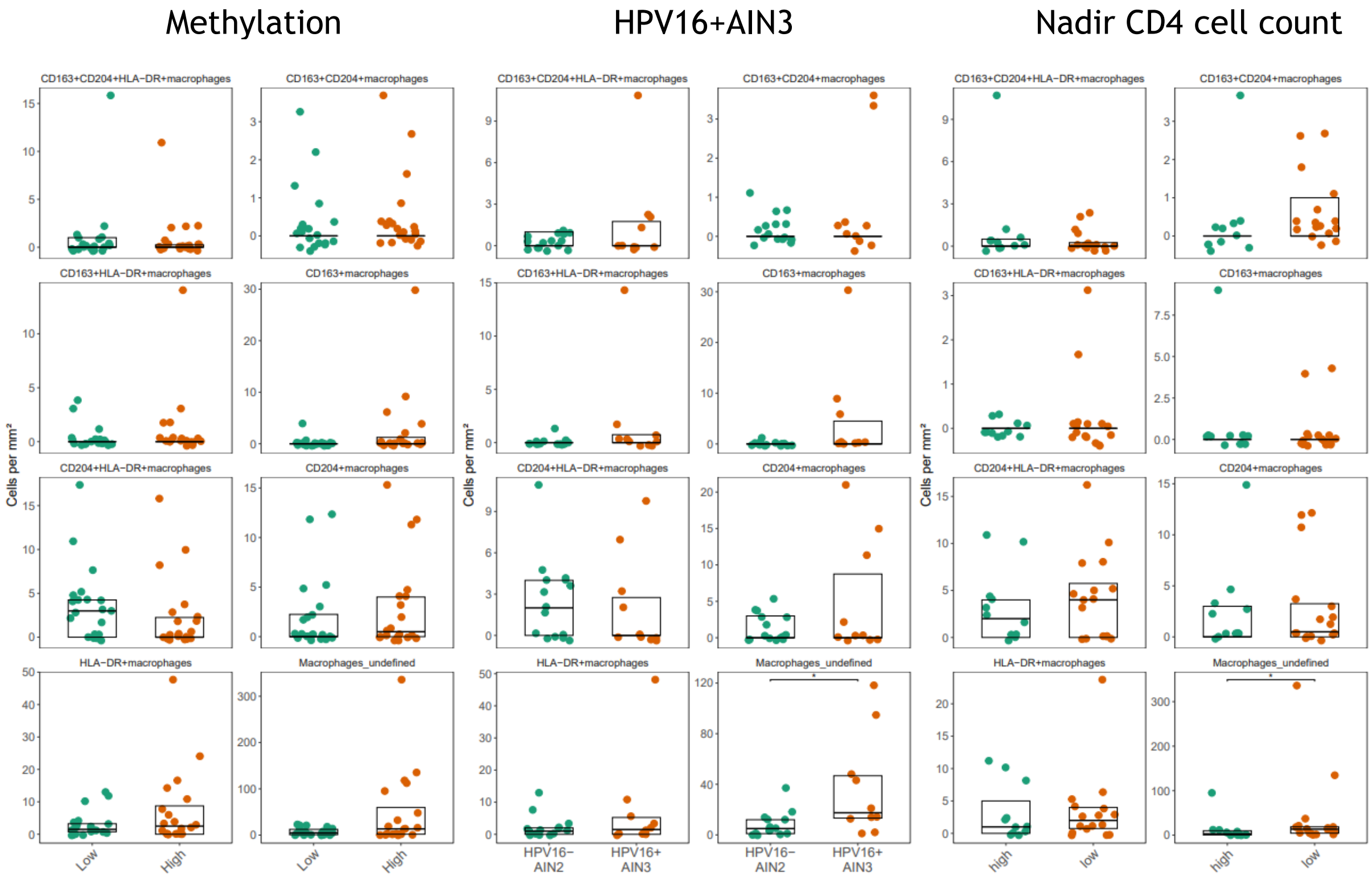

S18 Macrophage counts stratified by AIN grade

Macrophage densities in the across anal AIN grades measured by immunofluorescence. Each point shows area-normalized cell densities (cells per mm^2) for the different macrophage phenotypes within an individual biopsy. Point colors represent the disease categories (Control: grey, AIN2: green, AIN3: orange, and SCC: black). Cross-bars mark the median, with the tops and bottoms of the bars corresponding to the first and third quartiles, respectively.

*=*p*< 0.05; **=*p*<0.01; ***=p<0.001.

Abbreviations: HSIL: high-grade anal squamous intraepithelial lesions; AIN: anal intraepithelial neoplasia grade; SCC: squamous cell carcinoma.

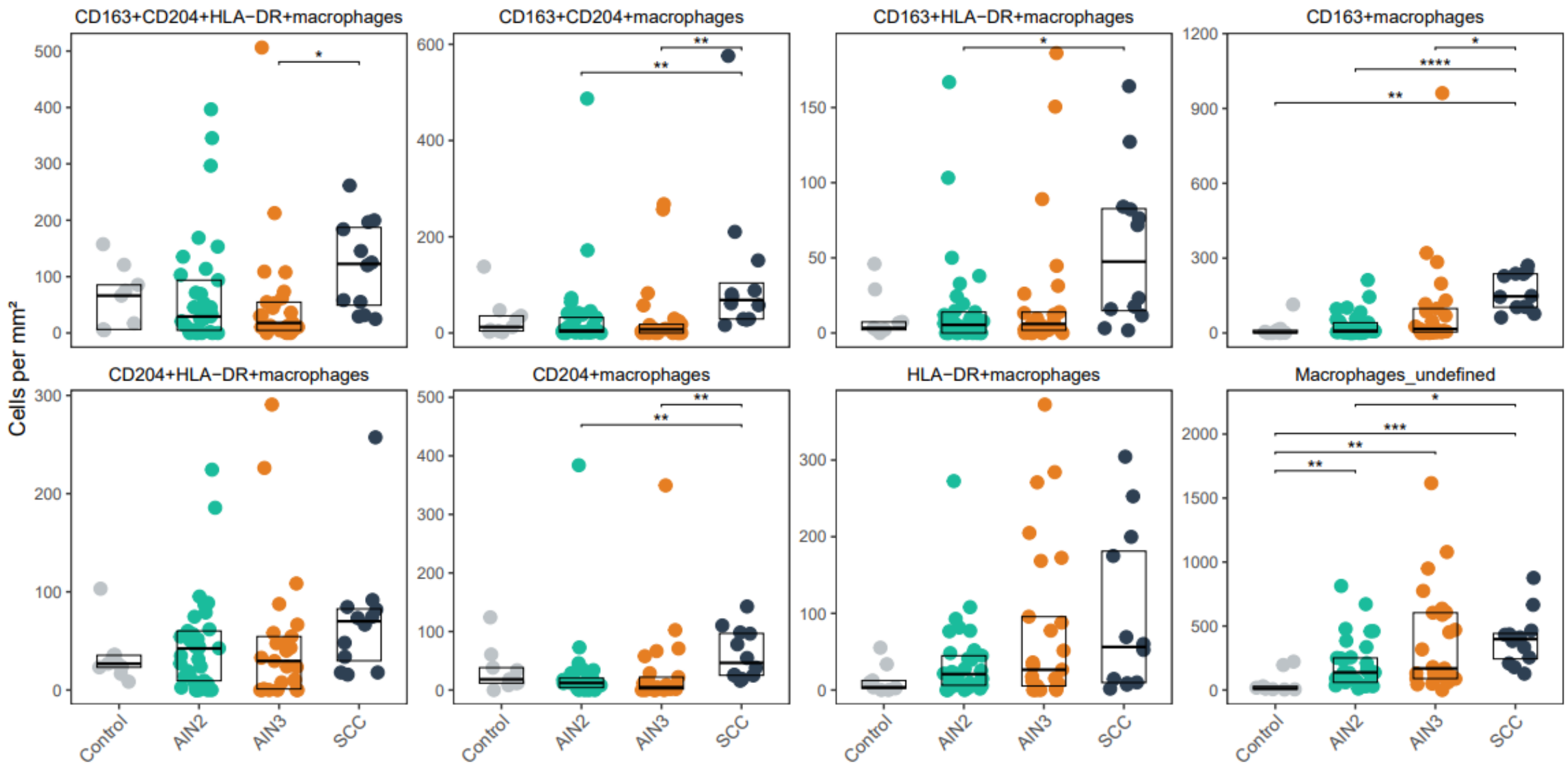

Supplementary Tables

Table S1: Antibody information imaging mass cytometry

Target	Clone	Metal	Dilution
**CD8a**	D8A8Y	146 Nd	50
**PD-1**	D4W2J	160 Gd	50
**ICOS**	D1K2T(tm)	161 Dy	50
**CD204**	J5HTR3	164 Dy	50
**CD103**	EPR4166(2)	168 Er	50
**Tbet**	4B10	170 Er	50
**CD19**	D4V4B	172 Yb	50
**CD163**	D6U1J	173 Yb	50
**TGFbeta**	TB21	115In	100
**HLA-DR**	TAL 1B5	141 Pr	100
**CD11b**	D6X1N	144 Nd	100
**Granzyme B**	D6E9W	150 Nd	100
**cleaved caspase**	5A1E	155 Gd	100
**CD39**	EPR20627	157 Gd	100
**VISTA**	D1L2G(TM)	158 Gd	100
**CD14**	D7A2T	163 Dy	100
**CD56**	E7X9M	167 Er	100
**CD7**	EPR4242	174 Yb	100
**CD11c**	EP1347Y	176 Yb	100
**TCRgd + 2nd Ab**	H41	148 Nd	50
**CD4 +2nd AB**	EPR6855	145 Nd	100
**CD45**	D9M8I	149 Sm	50
**CD3**	EP449E	153 Eu	50
**PD-L1**	E1L3N(R)	156 Gd	50
**FOXP3**	D608R	159 Tb	50
**CD27**	EPR8569	175 Lu	50
**Vimentin**	D21H3	194 Pt	50
**Keratin**	C11 and AE1/AE3	198 Pt	50
**B catenin**	D10A8	89Y	100
**CD20**	H1	142 Nd	100
**CD68**	D4B9C	143 Nd	100
**CD31**	89C2	147 Sm	100
**CD57**	HNK-1 / Leu-7	151 Eu	100
**Ki-67**	8D5	152 Sm	100
**P16ink4a**	D3W8G	154 Sm	100
**IDO**	D5J4E(TM)	162 Dy	100
**CD45RO**	UCHL1	165 Ho	100
**D2-40**	D2-40	166 Er	100

**CD38**	EPR4106	169 Tm	100
**CD15**	MC480	171 Yb	100
**Histone H3**	D1H2	209	50

Table S2: Antibody information immunofluorescence

**Marker**	**Marker concentration**	**Opal type**	**Opal concentration**
**CD68**	500	520	100
**CD163**	500	570	100
**HLADR**	500	620	400
**Keratin**	2000	650	400
**CD204**	500	690	100
